# Microbial communities and metabolic functions vary with spatial heterogeneity in cold-seep carbonates

**DOI:** 10.1093/ismeco/ycaf232

**Published:** 2025-12-11

**Authors:** Manman Ma, Minxiao Wang, Yue Liang, Yang Guo, Huan Zhang, Lei Cao, Lulu Fu, Gaowei Hu, Chengfeng Li, Thomas Mock, Chaolun Li

**Affiliations:** Institute of Oceanology, Chinese Academy of Sciences, Qingdao 266071, Shandong Province, China; Institute of Oceanology, Chinese Academy of Sciences, Qingdao 266071, Shandong Province, China; South China Sea Institute of Oceanology, Chinese Academy of Sciences, Guangzhou 510301, Guangdong Province, China; Institute of Oceanology, Chinese Academy of Sciences, Qingdao 266071, Shandong Province, China; Institute of Oceanology, Chinese Academy of Sciences, Qingdao 266071, Shandong Province, China; School of Environmental Sciences, University of East Anglia, Norwich Research Park, Norwich NR4 7TJ, Norfolk, United Kingdom; Institute of Oceanology, Chinese Academy of Sciences, Qingdao 266071, Shandong Province, China; Institute of Oceanology, Chinese Academy of Sciences, Qingdao 266071, Shandong Province, China; Institute of Oceanology, Chinese Academy of Sciences, Qingdao 266071, Shandong Province, China; Key Laboratory of Gas Hydrate, Ministry of Natural Resources, Qingdao Institute of Marine Geology, Qingdao 266237, Shandong Province, China; Laboratory for Marine Mineral Resources, Qingdao Marine Science and Technology Center, Laoshan Laboratory, Qingdao 266237, Shandong Province, China; Key Laboratory of Gas Hydrate, Ministry of Natural Resources, Qingdao Institute of Marine Geology, Qingdao 266237, Shandong Province, China; Laboratory for Marine Mineral Resources, Qingdao Marine Science and Technology Center, Laoshan Laboratory, Qingdao 266237, Shandong Province, China; Institute of Oceanology, Chinese Academy of Sciences, Qingdao 266071, Shandong Province, China; State Key Laboratory of Microbial Technology, Shandong University, Qingdao 266237, China; Institute of Oceanology, Chinese Academy of Sciences, Qingdao 266071, Shandong Province, China; South China Sea Institute of Oceanology, Chinese Academy of Sciences, Guangzhou 510301, Guangdong Province, China; College of Marine Sciences, University of Chinese Academy of Sciences, Beijing 100049, Beijing Municipality, China

**Keywords:** cold seep, carbonates, microbial communities, anaerobic oxidation of methane, metagenomics, biogeochemical cycles

## Abstract

Cold-seep carbonates, formed through interactions among methane, fluid chemistry, and microbial chemosynthesis, represent biodiversity hotspots in the deep sea. Spatial heterogeneity within these carbonates arises from variations in methane flux, yet the microbial contributions to this heterogeneity remain underexplored. Here we combined remotely operated vehicle-based *in situ* measurements, X-ray imaging, metagenomics, qPCR, and ^13^C-CH_4_ stable-isotope labeling to investigate microbial communities across carbonate habitats in the South China Sea. We found that methane flux linked to carbonate structural properties, shapes microbial metabolic interactions, notably anaerobic methane oxidation coupled with aragonite and FeS precipitation. These processes may contribute to self-sealing carbonate features, potentially reducing methane permeability and influencing geochemical gradients and geomorphology. Our findings reveal that microbiomes and their feedbacks play a significant role in shaping habitat-scale spatial heterogeneity of cold-seep carbonates, improving our understanding of methane cycling and carbonate ecosystem dynamics.

## Graphical Abstract

**Graphical Abstract:**
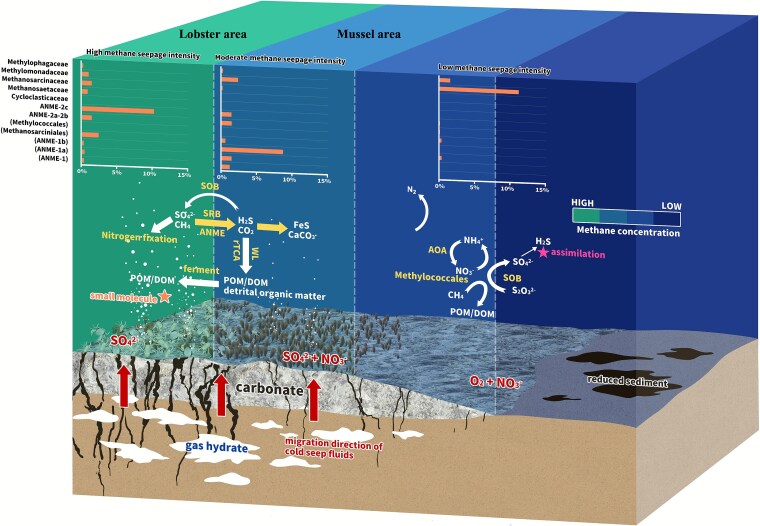

## Introduction

Cold seeps are chemosynthetic ecosystems in the deep sea, typically located along active and passive continental margins and subduction zones. These ecosystems are fueled primarily by methane, a key reductive molecule [1]. Microbial communities in cold seeps, mainly occupying sediments and the benthic boundary layers, can sequester ~80% of the methane, thus, playing a pivotal role in methane cycling and elemental dynamics [2, 3]. Research on cold-seep microorganisms has largely focused on sediments and seawater habitats, examining microbial diversity, distribution patterns, methane oxidation pathways, general metabolism, and their ecology [4–9]. For instance, in cold-seep ecosystems, intricate ecological networks optimize carbon, nitrogen, and sulfur cycling while enhancing methane filtration capabilities [9, 10]. In reduced sediments, co-occurring networks of anaerobic methane-oxidizing archaea (ANME) and Sulfate-reducing bacteria (SRB) dominate sediment communities, with distinct ANME–SRB microbiome formations according to the intensity of the methane seepage [8]. In bottom seawater of cold seeps, Methylococcus is present and facilitates a tight coupling of carbon, nitrogen, and sulfur cycles [9, 11].

However, an important geomorphological feature of cold seeps has received relatively less attention: carbonate formations. Usually, they cover active seepage regions, which significantly mediate the methane flux [12–14], microorganisms have been shown to play a crucial role in this regulation [15]. Case *et al*. [16] demonstrated that these carbonate environments host dynamic and diverse microbial communities. At the cold seep, anaerobic oxidation of methane (AOM) coupled to sulfate reduction elevates alkalinity and dissolved inorganic carbon (DIC), driving carbonate precipitation. Under strong AOM and high supersaturation, aragonite and high-Mg calcite commonly form; as seepage weakens and diagenetic evolution, the assemblage shifts toward low-Mg calcite and may undergo silicification, and the AOM-derived carbon imparts strongly negative δ^13^C signatures to the carbonates [17, 18]. Mineral phases and isotopes record and reflect the changes in precipitation-diagenesis and fluid supply [19]; aggregations of macrofauna, including lobsters and mussels, are typically co-occurring with active seepage and exhibit a patchy distribution within cold-seep habitats that reflects spatial variation in seep methane concentrations [20, 21]. In this habitat, interfacial chemical gradients (e.g. O_2_, NO_3_^−^, and CH_4_) exhibit reproducible spatial patterns [22, 23]. Taken together, current evidence indicates that seepage intensity is the primary determinant of spatial heterogeneity in carbonate habitats. By modulating carbonate supersaturation and interfacial redox conditions, it drives the zonation of mineral phases and regional macrofauna aggregations. We therefore define the recurring differences observed along the seepage gradient in mineralogy and pore architecture including their interfacial chemical and geological parameters as habitat-scale heterogeneity, which provides the framework for comparative analyses of associated microbial communities. Yet, data on how their habitat-scale spatial heterogeneity shape these microbial communities remains elusive. Based on what we have learned by studying microbiomes from sediments and seawater of cold seeps, it is likely that these carbonate microenvironments produce corresponding differences in the composition and function of resident microbial communities. Thus, here we test whether such heterogeneity exists, try to delineate its metabolic underpinnings, and evaluate how it may structure species interactions. The complex geochemical environments of the cold-seep carbonate make it an appropriate laboratory to study the metabolic interactions and their influence on methane sequestration.

In order to address the knowledge gap in terms of the role of cold-seep carbonate environments in shaping microbiomes, we have studied an active seepage location in the South China Sea (F site), characterized by extensive carbonate outcrops and dense biological communities [24]. This carbonate-covered zones exhibit significant heterogeneity in terms of megafauna covered and interface geochemical conditions. Hence, this location is ideal to shed light on links between spatial heterogeneity and the activity and diversity of associated microbiomes. Central regions of the F site, characterized by dense lobster beds, experience the highest intensity of methane seepage, while adjacent mussel beds have slightly lower methane seepage. Peripheral regions are lacking typical chemosynthetic communities and only have low methane seepage activity [14, 23, 25]. These observations indicate that these carbonates may differ in pore connectivity, methane flux, and their related properties. We therefore have analyzed how these differences affect microbial communities and functions. The main outcome of our work revealed that the methane flux linked to carbonate structural properties, shapes microbial metabolic interactions, notably anaerobic methane oxidation coupled with aragonite and FeS precipitation. These processes may contribute to self-sealing carbonate features, potentially reducing methane permeability and influencing geochemical gradients and geomorphology.

## Materials and methods

An overview of the study design and workflow is provided in Fig. S1. The following sections describe the details of the material and methods used in this study.

### Sampling

**Figure 1:**
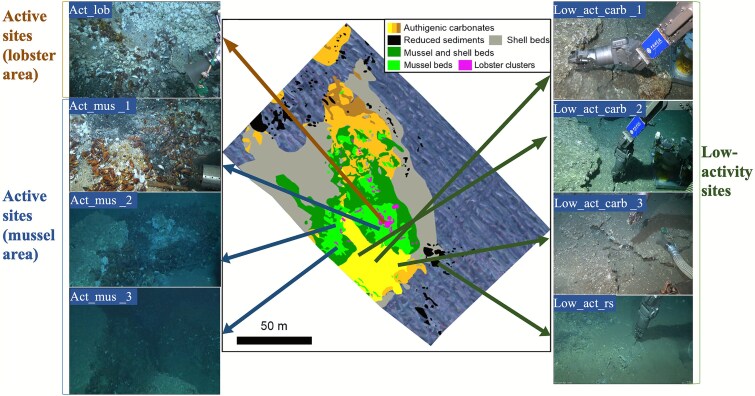
Geomorphology of the collection site for carbonate samples at the cold seep (site F).

Eight carbonate samples (Fig. S2) were collected in 2021 from Site F, northern South China Sea, using the remotely operated vehicle (ROV) Faxian aboard R/V *Kexue*. Samples were recovered in a thermally insulated biobox to prevent seawater contamination. Based on seabed characteristics, four distinct habitats were identified: clusters of squat lobsters around seep vents, extensive mussel beds, exposed carbonate zones lacking megafauna, and peripheral areas with reduced sediment cover [14]. Chemosynthetic invertebrate communities varied with methane concentration, as observed *in situ* and corroborated by previous studies [14, 23]. As in prior work [16, 26], active sites included areas densely populated by *Shinkaia crosnieri* lobsters (Act_lob) and *Gigantidas platifrons* mussels (Act_mus), while low-activity sites featured bare carbonate areas (Low_act_carb) and reduced sediment zones (Low_act_rs), as depicted in Fig. 1. Data from integrating interface geochemistry (CH_4_ and O_2_), carbonate structure (porosity and pore connectivity), and chemosynthetic megafauna as seep-intensity indicators, corroborated previous work [14, 23, 27, 28]. Stronger upward seepage yields higher methane flux and, therefore, supports a proxy-based ranking of methane flux regimes from high to low: Act_lob (high) > Act_mus (intermediate) > low_act_carb/low_act_rs (low). Upon retrieval, samples were immediately stored in sterile bags at −80°C. For laboratory incubation experiments, carbonate (Act_mus, Low_act_carb) and surface reduced sediment samples were collected and transported to the laboratory in vacuum-sealed containers at 4°C. Additionally, temperature, dissolved oxygen (DO), and CH₄ concentrations at the seabed and carbonate interfaces were measured *in situ* using active sensors on ROV *Faxian* [23]. Nitrate (NO₃^−^) concentrations at the interface were determined using standard colorimetric methods with a continuous flow analyzer (AutoAnalyzer 3, Seal Analytical Ltd., UK) [9].

### Physical and chemical characterization of carbonates

The phoenix v|tome|x industrial computed tomography was utilized to acquire high-resolution 2D images of pores and fractures in the carbonate samples. These images facilitated 3D model reconstruction for analyzing the spatial distribution, connectivity of pores, fractures, and overall porosity. X-ray diffraction analysis was performed using a D8 ADVANCE diffractometer with CuKα radiation (40 kV, 40 mA) at the Institute of Oceanology, Chinese Academy of Sciences (Qingdao). The analysis employed a step frequency of 0.5 s/step across a scanning range of 5°–75° (2θ). Carbon and oxygen isotopic compositions were measured using a MAT 253 Stable Isotope Ratio Mass Spectrometer (Thermo Fisher Scientific, USA) at the Yantai Institute of Coastal Zone Research, Chinese Academy of Sciences. Carbon isotope ratios were referenced against the VPDB standard and oxygen isotopes were benchmarked against the Vienna Standard Mean Ocean Water standard.

### DNA extraction and qPCR analysis

To minimize contamination risks, only the inner portions of the carbonate rocks were used for DNA sampling for the main dataset. Approximately 4 × 4 × 4 cm samples were cut from the geometric center of irregular carbonates and ground with a sterile mortar. Separately, the samples of the surface layers (~0.4 cm thick, marked with “S”) were thoroughly rinsed with sterile water, and ground to investigate microbial biofilms or surface-associated biogeochemical processes potentially influencing carbonate dynamics. DNA was extracted from 300 to 500 mg of powdered rock using the DNeasy PowerLyzer PowerSoil Kit (Qiagen), following the manufacturer’s protocol.

For qPCR analysis, the QuantStudio™ 1 System (ABI, USA) and Fast Start Universal SYBR Green Master (Rox) Kit (Roche, Switzerland) were used. The *mcrA*, *pmoA*, *dsrB*, and *nirS* genes were amplified using primers from prior studies as listed in Table S1 [29–31]. The reaction mixture included 1 μl template DNA, 10 μl Rox, 1 μl each of forward and reverse primers (10 μM), and 7 μl ddH₂O. Amplification involved initial denaturation at 50°C for 2 min, 95°C for 10 min, followed by 40 cycles of 95°C for 15 s and 54°C for 2 min. Negative controls were included for quality assurance, producing no positive signals. The construction of standard curves is described in the supplementary methods, and the resulting standard curves are shown in Fig. S3.

### Metagenomic sequencing and data processing

For metagenomic library preparation, ~100 ng of DNA was fragmented to a size range of 500–800 bp using a Covaris E220. Libraries were sequenced on the DNBSEQ platform using paired-end reads of 100 bp at BGI. Raw data are available under NCBI SRA project PRJNA1061233. Sequence quality control was performed with Fastp [32].

Microbial species were identified from metagenomic shotgun data using *phyloFlash* [33], which reconstructs and classifies SSU rRNA sequences, utilizing the SILVA database (version 138.1) for taxonomic assignment. Assembly and gene prediction employed Megahit [34] and Prokka [35], respectively. Microbial co-occurrence networks were constructed via sparCC algorithm [36]. Genes were annotated against the KEGG database using Diamond [37]. Gene abundance was estimated with Salmon [38] in mapping-based mode, enabling GC-bias and sequence bias correction. Expression levels were calculated as Genes Per Million (GPM) to normalize for gene length and sequencing depth, thus, facilitating cross-sample comparisons. No external reference sample was used. Key gene statistics were assessed by two-sided Fisher’s exact test with false discovery rate (FDR) correction (STAMP v2.1.1) [39]. Metawrap processes [40], including METABAT, MAXBI,N, and CONCOCT, were used for binning. Metagenome-assembled genomes (MAGs) with a completeness rate <70% or contamination >10% were excluded, as assessed by CheckM [41]. Taxonomic identification of MAGs was performed with GTDB-tk [42]. The completeness of metabolic modules in each MAG was assessed based on the presence of module-defining KEGG Ortholog (KO) genes (supplementary module_completeness.py). Model-predicted metabolic exchanges (from SMETANA applied to MAG-derived GEMs) are provided as supporting information (Supplementary Methods and Supplementary Results).

### Isotope-labeled cultivation experiment

Refer to Marlow *et al*. [15], laboratory cultivation was performed using carbonate samples from the active (Act_mus) and low-activity sites (Low_act_carb), and surface reduced sediments. Samples were sectioned into ~1 cm^3^ blocks and incubated for 9 h at 10 MPa (simulating deep-sea pressure) and 4°C using δ^13^C-CH_4_. Each incubation consists of 10 ml of carbonate or sediment and 80 ml of bottom seawater. The experiment included six groups: sediment samples under aerobic and anaerobic conditions, active site carbonate samples [aerobic (CAE_act) & anaerobic (CAN_act) conditions], low-activity site carbonate samples [aerobic (CAE_low_act) & anaerobic (CAN_low_act) conditions]. All culture media in high-pressure vessel were precooled at 4°C. After introducing the sample, the headspace was quickly pressurized to 10 MPa (within ~10 min), and incubation commenced immediately without additional equilibration time. During postincubation, DNA was extracted directly from samples of the incubated carbonate or sediment material. 16S rRNA gene sequencing was performed, and ^13^C-DIC values were measured using a Delta V Advantage isotope ratio mass spectrometer coupled with a GasBench II system, equipped with a PoraPlot Q chromatography column (30 m × 0.32 mm × 20 μm).

## Results

### Geochemical conditions of the sampling sites

Act_lob samples were collected from a carbonate zones covered by dense squatter lobster area with high methane (8.1 μM), low DO (0.1–1.5 mg/l), and relatively low nitrate (17.37 μM) at the interface compared to surrounding regions (Table S2). Nearby, the Act_mus region, carbonates covered by dense mussel beds, showed decreased methane (1.6 μM) and increased oxygen (0.8–3.1 mg/l) and nitrate (22.32 μM). The low_act_carb region, on the outskirts of the F site, had the lowest methane (0.4 μM), accompanied by increased oxygen (>2.5 mg/l) and nitrate levels (24.2 μM).

### Mineral heterogeneity of carbonate

**Figure 2:**
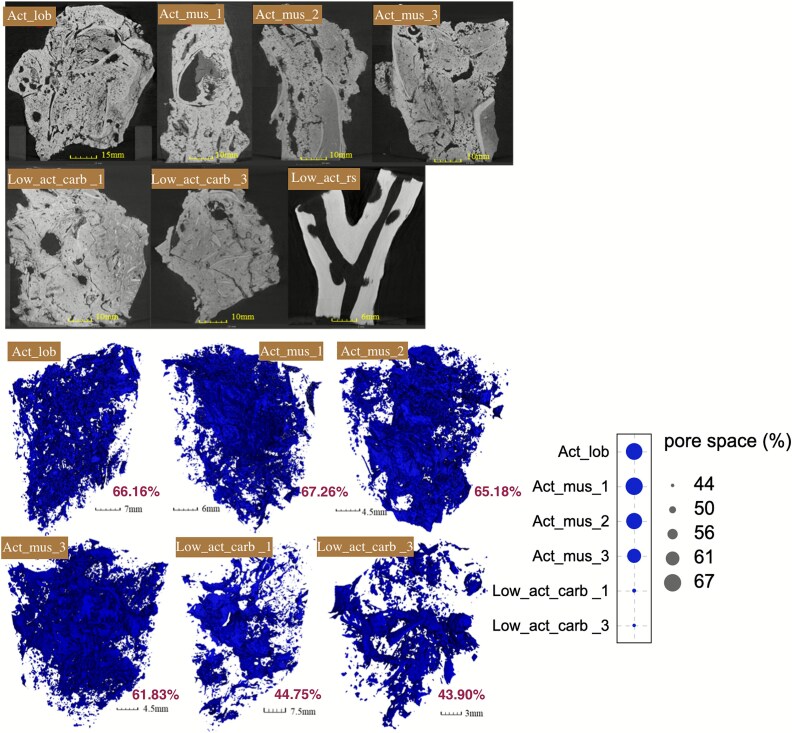
2D and 3D pore distribution in carbonate samples. In the 2D diagram, grayish white represents the carbonate, and the black in the rock represents the pores. In three dimensions, blue represents the pores.

Carbonate samples from different environments exhibited distinct mineralogy and internal structure (Fig. 2). Carbonates from active sites mainly comprised aragonite (84%–89%), embedded with biological shells, and exhibited high porosity (9.56%–11.56%, Table S2), coarse pores, cracks, and dense micropores, forming interconnected networks (Fig. 2). In contrast, low_act_carb samples demonstrated lower porosity (6.01%–9.18%) and reduced pore connectivity (Fig. 2). Despite limited macrofauna (Fig. 1), abundant biological shells (Fig. S3) and high aragonite content (84%) indicated previous cold-seep activity. The low_act_rs tubular sample, collected from reduced sediment at the interface, consisted mainly of calcite (98%) and had a carbon isotopic value (−3.32‰) suggestive of abiotic carbon sources.

### Microbiome community structure of carbonate samples based on PhyloFlash

**Figure 3:**
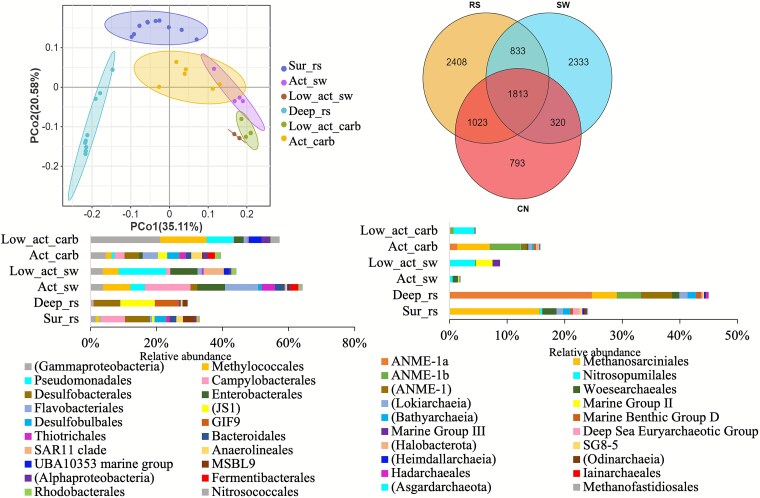
Principal coordinates analysis (PCoA) diagram of communities by phyloFlash analysis in different habitats; Venn diagram of microbial distribution in different habitats; bacteria (left) and archaea (right) composition in different habitats at the order level. Sur_rs: 0–20 cm of reduced sediments; Deep_rs: 20–40 cm of reduced sediments; Act_sw: Bottom seawater of active site; Low_act_sw: bottom seawater of low_active sites; RS: reduced sediments; SW: bottom seawater; Carb: carbonate.

PhyloFlash analysis identified 6077, 5299, and 3949 reconstructed SSU rRNA sequences from reduced sediment, bottom seawater, and carbonate samples, respectively (Fig. 3B). PCoA of community composition (PC1 = 35.11%, PC2 = 20.85%) separates reduced sediment from seawater and carbonate samples, while seawater and carbonate samples occupy a partially overlapping space that further diverges by activity level (Fig. 3A). In active sites, seawater overlaps only partially with carbonate samples; in low-activity sites, seawater plots closer to carbonate samples, indicating stronger microbial community similarity at low activity than at high activity. The microbial overlap at low activity is driven by archaeal Nitrosopumilales together with bacterial Methylococcales, Pseudomonadales, and Enterobacterales, whereas at active sites the shared signal is associated with Campylobacterales, Flavobacteriales, Thiotrichales, Fermentibacterales, and Desulfobacterales (Fig. 3C and D). Besides, carbonate habitats harbored distinct taxa such as ANME–SRB, which were also prevalent in sediments. Habitat-specific microbes in carbonate samples were scarce and mainly included low-abundance taxa such as *IheB2–31* (Thioglobaceae), *Burkholderiales SC-I-84*, *Porphyromonas* sp. (*COT-290 OH860*), and *Enterobacter cloacae*, with relative abundances <0.006% of the total estimated community.

Linear discriminant analysis effect size (LEfSe, LDA >3, Fig. S4) revealed significant enrichment of ANME-2c, ANME-1b, SRB, Methylophagaceae, Anaerolineaceae, Kiloniellales, and Pirellulaceae in carbonate habitats. Microbial communities in carbonates clustered distinctly by sampling locations (ANOSIM, *P* = .002; Fig. S5).

Compared across regions (Fig. S5), carbonates of Act_lob group (Act_lob and Act_lob_s) were dominated by ANME-2c (10.15% ± 3.15%), Desulfosarcinaceae (6.55% ± 2.72%), Desulfobulbaceae (4.68% ± 0.76%), Fermentibacteraceae (8.05% ± 3.03%), Flavobacteriaceae (4.42% ± 0.60%), and Calditrichaceae (4.05% ± 0.36%). In contrast, Act_mus samples (Act_mus_1, Act_mus_1_s, Act_mus_2, Act_mus_3) were dominated by ANME-1b (8.05% ± 4.94%) rather than ANME-2c and showed lower Desulfosarcinaceae (2.80% ± 1.64%), with additional contributions from Desulfocapsaceae (1.37% ± 0.71%), Desulfatiglandaceae (1.22% ± 0.80%), Flavobacteriaceae (4.05% ± 3.39%), Anaerolineaceae (2.84% ± 2.22%), Sulfurovaceae (2.42% ± 0.75%), and Thiotrichaceae (2.33% ± 2.02%). Low-activity samples differed from active site samples, being dominated by Methylomonadaceae (11.18% ± 7.41%), Thioglobaceae (5.56% ± 3.85%), and Nitrosopumilaceae (4.05% ± 1.00%), with additional families including Kiloniellaceae (3.01% ± 1.51%), Pirellulaceae (1.57% ± 1.11%), Methylophagaceae (1.51% ± 1.60%), Woeseiaceae (1.23% ± 0.72%), and Enterobacteriaceae (1.22% ± 0.14%).

### Metabolic functions derived from metagenomes

**Figure 4:**
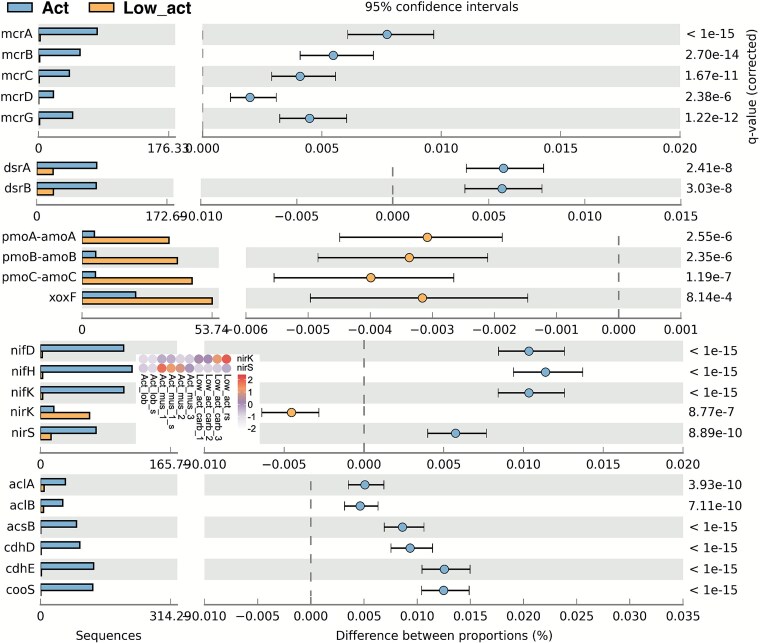
Differences in the distribution of metabolic genes involved in methane oxidation (*mcrA/B/C/D/G*, *pmoA/B/C*), sulfate reduction (*dsrA/B*), nitrogen fixation (*nifD/H/K*), denitrification (*nirK/S*) and carbon fixation (*aclA/B*, *acsB*, *cdhD/E*, *cooS*) between active and low_activity samples. The *q* values are based on Fisher’s exact test and corrected by Benjamini–Hochberg FDR using STAMP.

Approximately 25 million non-redundant genes, comprising a gene catalog clustered at 95% nucleotide identity, were derived from all carbonate samples, encompassing key genes involved in carbon, sulfur, and nitrogen metabolism (Fig. 4). The samples predominantly clustered into two groups based on their relative gene abundances quantified as GPM: active and low active sites in terms of methane flux (Fig. S6).

#### Aerobic and anaerobic methane oxidation

Genes for anaerobic methane oxidation (*mcrA/B/C/D/G*) were at least 32-fold more abundant in active sites (Fisher’s exact test and corrected by Benjamini–Hochberg FDR, *P <* 10^−6^; Fig. 4). Conversely, abundances of aerobic methane oxidation genes (*pmoA* and *xoxF*) were significantly higher in low-activity sites (at least 3-fold changes, *P <* 10^−4^). qPCR supported these trends, with *mcrA* gene abundance peaking at ~10^8^ copies/g in active sites versus 5.65 × 10^5^ copies/g in low-activity sites (Fig. S7). While the *pmoA* gene increased from 1.30 × 10^5^ copies/g at the low-active sites to 1.41 × 10^7^ copies/g at the most active methane seepage sites.

#### Carbon fixation

Genes underpinning the reductive tricarboxylic acid (rTCA) cycle and the Wood–Ljungdahl (WL) pathway were on average 30-fold more abundant at active sites (*P* < 10^−10^; Fig. 4). A substantial proportion of these genes was attributed to SRB, including 30.69% of *korA* and 33.57% of *korB* for rTCA, and 20.77% of *cdhD* and 38.71% of *cdhE* for WL. Similarly, a notable share was assigned to ANME, accounting for 46.38% of *cdhD* and 42.63% of *cdhE* for WL. Additionally, these genes exhibited a significant positive correlation with *mcr* genes (Pearson, *P* < .05).

#### Nitrogen metabolism

Nitrogenase subunit genes *(nifD/K/H*) and *hao*, which are associated with nitrogen fixation and nitrification, showed at least 34-fold higher abundance at active sites (*P* < 10^−15^; Fig. 4). Most of these nitrogen fixation genes (32.75%–47.13%) were taxonomically assigned to ANME–SRB consortia based on eggNOG annotation. Furthermore, these genes demonstrated a significant positive correlation with *mcr* genes (*P* = 4.27 × 10^−9^). Genes related to denitrification were 2-fold more abundant in regions with low to moderate methane flux (act_mus and low_act; Fig. 4), including *nirS*, which showed an abundance an order of magnitude higher than that measured at the lobster site (Fig. S7).

#### Sulfur metabolism

Genes encoding adenylylsulfate reductase (*aprA*) and sulfite reductase (*dsrA/B*) were 35% more abundant at active sites (*P* < .024; Fig. 4), and positively correlated with *mcr* genes, nitrogen fixation genes (*nifH* and *nifK*), and carbon fixation genes (*oorA*, *oorB*, *oorC*, and *oorD*). qPCR confirmed that the *dsrB* gene reached its highest abundance at 9.89 × 10^7^ copies/g in active-site samples, with a gradual decline across the lobster, mussel, and low-activity areas (Fig. S7).

For the Sox sulfur oxidation system, *soxA, soxB, and soxX* were more abundant in active sites, although the differences were not statistically significant (*P* > .081). Conversely, *soxC, soxD, soxY*, and *soxZ* were more prevalent in low-activity samples. Genes encoding sulfide-quinone oxidoreductase (*sqr*) were also more abundant in active sites.

#### Fermentation

Analysis of carbohydrate-active enzymes revealed widespread presence of enzymes related to carbohydrate metabolism across carbonate habitats (Fig. S8). These enzymes were more abundant at the active site and displayed the potential to degrade a diverse array of carbohydrates, including chitin, cellulose, pectin, polyphenolics, starch, and xyloglucan (Table S3).

### Co-occurrence of microorganisms

#### Microbial co-occurrence networks

**Figure 5:**
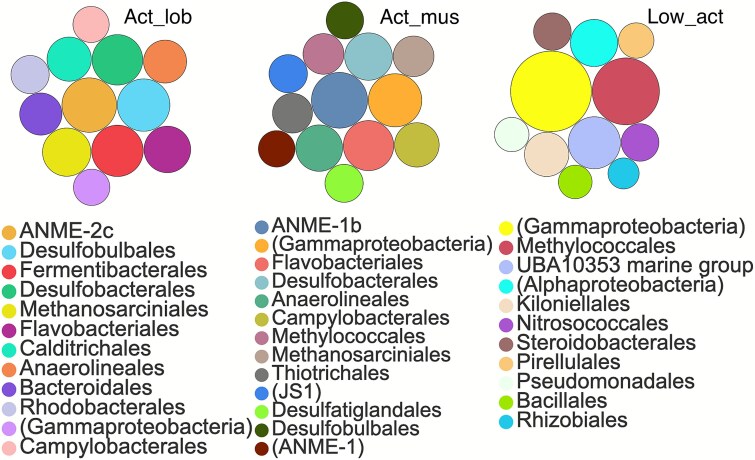
The taxonomy in major modules of network analysis in different regions.

Network analysis was performed to explore co-occurrence patterns and potential interactions among microbial taxa across different sampling areas. The SSU rRNA sequences were grouped into three main modules (Fig. S9). Graphs 2 and 3 accounted for 44% and 53.95% of total relative abundance, respectively (Fig. S10). ANME-2c was abundant in the lobster area, co-occurred with SRB, Fermentibacterales, Anaerolineales, and Calditrichales. In mussel area, ANME and SRB, particularly ANME-1b, were the dominant co-occurring taxa (Fig. 5). These taxa were positively correlated with others, such as Campylobacterales, Thiotrichales, Fermentibacterales, and Anaerolineales. In contrast, Methylococcales, primarily found in low-activity areas, co-occurred with UBA10353 marine group, Nitrosococcales, and Kiloniellales. These findings highlight distinct microbial co-occurrence patterns across sites and underscore the variability of taxa interactions in cold-seep ecosystems.

Emerging evidence highlights the significant role of biofilms in shaping co-occurring microbial communities. Among the annotated gene sets, 187 genes linked to biofilm formation—such as c*rp, csrA, glgA,* and *glgC*—and 152 genes associated with quorum sensing, including *trpE* and *ACSL*, were highly abundant (Fig. S10).

### Metabolic reconstruction of MAGs

A total of 434 MAGs were selected based on a completeness threshold >70% and contamination levels <10%, resulting in 341 non-redundant MAGs (Fig. S11), which cover 14% of the reads sequenced. These MAGs were taxonomically classified into 40 phyla.

**Figure 6:**
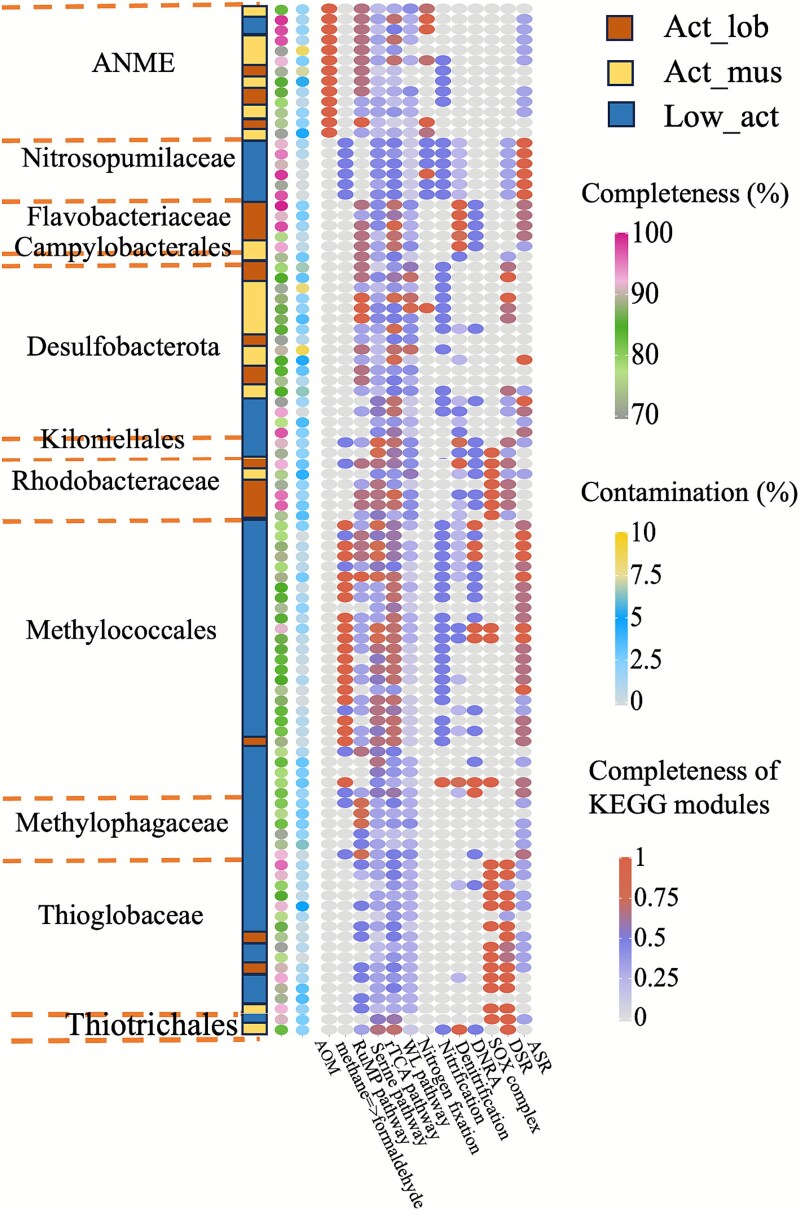
Carbon, nitrogen, and sulfur metabolic capacities for the dominant MAGs. RuMP pathway: M00345 Formaldehyde assimilation, ribulose monophosphate pathway; DSR: M00596 Dissimilatory sulfate reduction, sulfate = > H_2_S; ASR: M00176 Assimilatory sulfate reduction, sulfate = > H_2_S.

ANME–SRB consortia mediate AOM and fix carbon via the rTCA and/or WL pathways, and they commonly encode nitrogenase, indicating capacity for N_2_ fixation that can alleviate nitrogen limitation (Fig. 6). Aerobic methanotrophs (Methylococcales and Methylophagaceae) oxidize methane/methanol via pmo and xoxF and assimilate carbon through the RuMP pathway; a fraction of formaldehyde is further oxidized via the H_4_MPT route to CO_2_, potentially driving mild acidification under weak buffering (Fig. 6). Heterotrophic fermenters (Fermentibacteraceae, Calditrichaceae, Flavobacteriaceae, and Anaerolineaceae) encode broad carbohydrate hydrolases (Fig. S12) and pta–ackA or acs together with hydrogenases (*hydA/hydB, mbhJ,* and *echE*), enabling production of H_2_ and acetate; some Flavobacteriaceae appear mixotrophic and harbor rTCA genes, and in act_mus samples they additionally possess gene sets consistent with a complete denitrification pathway. Methylococcales, Rhodobacteraceae, Campylobacterota, and Thiotrichales encode sqr and a complete SOX system, whereas Thioglobaceae lack *soxC/D* but utilize *soxA/B/X/Y/Z* alongside a complete dsr module; notably, methanotrophic Methylococcales include seven genomes with *dsrA/B*, and three carry complete SOX systems (Fig. S13). At low-activity sites, Nitrosopumilaceae harbor *amoA/B/C*, consistent with archaeal ammonia oxidation. In contrast to the act_mus samples, genes for denitrification and DNRA (dissimilatory nitrate reduction to ammonium) in this region were encoded by Methylococcales and Kiloniellales.

#### Potential metabolic interactions within the microbiomes

Our simulated metabolic interaction analysis revealed pronounced potential for microbial competition and cooperation across all samples (Fig. S14A and B). In carbonate habitats, a negative correlation was observed between model predicted metabolic resource overlap (MRO) and metabolic interaction potential (MIP), with a correlation coefficient of −0.911 (*P* < .05, Fig. S14C). However, no distinct regional patterns were identified in the spatial distribution of these interactions. Notably, the predicted cooperation potential, as inferred from genome-scale metabolic models, was higher in inner carbonate samples compared to bottom seawater, reduced sediments, and surface carbonate samples, suggesting that carbonate-associated microbes may efficiently utilize resources via predicted metabolite exchange through permeable junctions.

Further analysis of the predicted metabolite exchanges based on metabolic modeling indicated key microbial interactions. In active zones, the frequent potential exchange of nitrogenous compounds (Fig. S15), such as glutamine and amino acids (e.g. lysine and arginine), supported the hypothesis of tightly coupled carbon and nitrogen cycling. Complementing these community-level inferences, we examined putative receiver taxa (e.g. Anaerolineales). MAGs affiliated with Anaerolineales encoded the complete branched-chain amino acid ABC transporter (liv operon), including *LivF* (ATPase), *LivG* (ATPase), *LivH* (permease), *LivM* (permease), and *LivK* (substrate-binding protein). Consistent with the predicted amino-acid exchanges, all five genes showed consistently higher relative abundances in active sites than in low-activity sites (Fig. S16).

### Methane oxidation and ^13^C-DIC production under different environmental conditions

**Figure 7:**
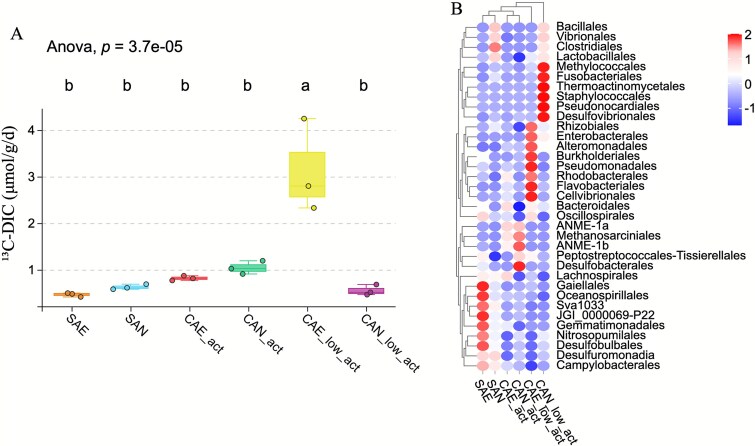
(A) Boxplots showing rates of ^13^C-labeled DIC production in different sample groups. Each box represents the interquartile range (IQR; 25th–75th percentile), the horizontal line indicates the median, whiskers indicate the minimum and maximum values within 1.5 × IQR, and individual dots represent biological replicates (*n* = 3). Letters above boxes indicate significance groups from Tukey’s post hoc test after one-way ANOVA; groups sharing the same letter are not significantly different (*P* > .05). (B) Heatmap showing relative abundance (z-score) of dominant microbial orders across the same sample groups.

Across the six incubation treatments, ^13^C-DIC production differed significantly overall (one-way ANOVA, *P* = 3.7 × 10^−5^; Fig. 7A). BH-corrected post hoc tests identified a single difference: carbonate from the low-activity site incubated under aerobic conditions (CAE_low_act) had higher rates than each of the other five treatments; all other pairwise comparisons were not significant. CAE_low_act ranged from 2.3 to 4.2 μmol g^−1^ d^−1^, whereas the remaining treatments clustered below 1.2 μmol g^−1^ d^−1^. Endpoint 16S rRNA profiles (Fig. 7B) are presented as relative compositions. At the low-activity site, aerobic incubations showed higher relative abundances of several heterotrophic orders (e.g. Rhizobiales, Enterobacterales, and Alteromonadales), whereas anaerobic incubations showed higher relative abundances of Methylococcales, Desulfovibrionales, and heterotrophic orders including Fusobacteriales, Thermoactinomycetales, and Staphylococcales. Endpoints from the active site were characterized by higher relative abundances of ANME-1 and Desulfobulbales.

## Discussion

**Figure 8:**
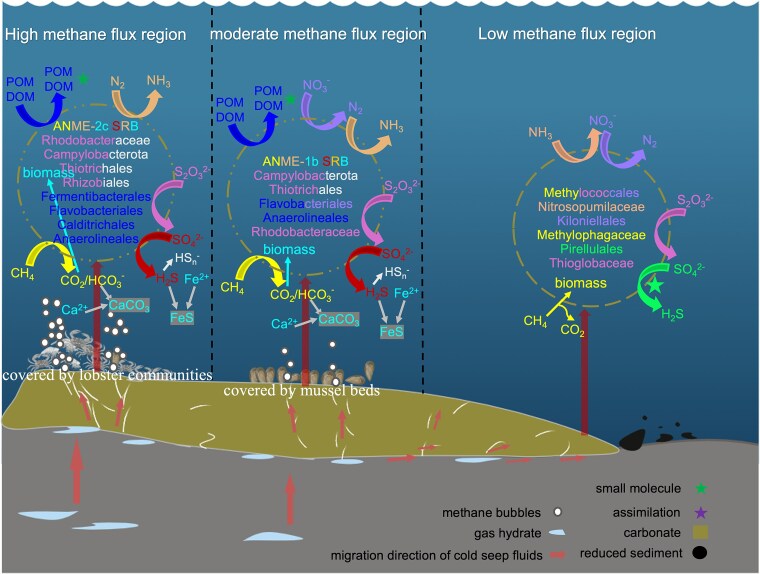
Microbial distribution and metabolism model in cold-seep carbonate habitats.

The continuous AOM facilitated by various ANME–SRB consortia appears to drive the formation of carbonates with diverse morphologies, porosity, and connectivity to aerobic seawater [14, 26]. Our results demonstrate that cold-seep carbonate habitats display pronounced heterogeneity across different sampling sites (e.g. Act_lob, Act_mus, and low_act_carb). This heterogeneity coincides with distinct gradients in methane, oxygen, and nitrate concentrations, as well as macrofauna distribution. These site-to-site differences are closely linked to the assembly of distinct microbial consortia and their specialized metabolic functions within each area, shaping both community structure and biogeochemical activity. Consistent with these environmental differences, carbonate communities separate by location and methane activity level (Fig. 8), with ANME–SRB consortia prevailing at active sites and oxygen/nitrate-linked assemblages (e.g. Methylococcales and Nitrosopumilales) more prominent at low-activity sites. Specifically, within active site carbonates, higher-methane zones (lobster area) were dominated by ANME-2c with Desulfosarcinaceae partners, whereas relatively lower-methane zones (mussel area) were enriched in ANME-1 (primarily ANME-1b) with Desulfobulbales. This partitioning aligns with prior reports that ANME-2c preferentially occupies high-methane niches [43, 44], supporting the suggested link between methane availability and community composition.

In AOM-active carbonates, gene inventories and MAG assignments support a tight coupling between methane oxidation and inorganic carbon assimilation. Heterotrophs and putative autotrophs (e.g. Rhodobacteraceae and Rhizobiales) co-occur with ANME–SRB and encode fixation pathways, suggesting a possible role in incorporating AOM-derived CO₂. Additionally, FeS (Fig. S17) and aragonite precipitation during AOM suggest self-sealing properties of the carbonate formations [14]. These transformations may enhance the containment of methane and suggest that high-methane flux regions are important sites for carbon cycling.

The potential for nitrogen cycling varies systematically with the environmental conditions of each site (Fig. 8) and are likely governed by a carbon–nitrogen imbalance [45]. At higher methane concentrations and in low-oxygen zones (e.g. lobster and mussel areas), nitrogenase genes (*nifH/D/K*) are more abundant and correlate with mcr, consistent with intensified carbon throughput and increased cellular nitrogen demand under O_2_-limiting conditions. This pattern aligns with the geochemical context (higher CH_4_, lower O_2_) and the known O_2_ sensitivity of the nitrogenase enzyme [45–49]. Notably, where nitrate is relatively high within the active site carbonates, gene abundance underpinning denitrification increased. For instance, Flavobacteriaceae MAGs include a complete denitrification pathway, consistent with nitrate-driven respiratory flexibility in these communities. In contrast, low-activity carbonates characterized by higher O_2_ and nitrate, show reduced potential for nitrogen fixation and increased abundance of nitrate reduction/denitrification genes, together with archaeal ammonia oxidation (Nitrosopumilaceae). This indicates a shift towards nitrate-dependent and aerobic nitrogen cycling. These findings suggest that nitrogen cycling in cold-seep carbonate habitats exhibits a high degree of plasticity and adaptability. Therefore, microbial communities appear to be able to flexibly adjust their metabolic pathways according to the availability of methane and nitrogen sources, thereby maintaining ecosystem functions.

For sulfur redox pathways, *dsrA/B* are more abundant at active methane sites and correlate with mcr as well as carbon and nitrogen fixation genes, which is consistent with sulfate dependent AOM. Sulfur oxidizing lineages vary by region (Fig. 8). For instance, in high-methane areas taxa such as Campylobacterota, Thiotrichales, and Rhodobacterales are present. By contrast, in low-methane areas, several Methylococcales MAGs encode *dsrA/B* together with a complete SOX system, indicating sulfur linked metabolic versatility under oxygenated or nitrate replete conditions [50–52]. Thioglobaceae in low-methane active carbonates lack *soxC/D* but encode *soxA/B/X/Y/Z* alongside a complete dsr module, consistent with a reverse-Dsr oxidative strategy [53–57]. Members of this group can utilize oxygen or nitrate as electron acceptors and may engage in cooperative sulfur cycling with denitrifying microorganisms [53, 55]. Together, these observations support lithology and geochemistry dependent coupling of carbon fixation with nitrogen and sulfur transformations across carbonate habitats.

Co-occurrence patterns and MAG-derived metabolism indicate distinct microbial interactions across different sites. Methane active site carbonates were dominated by specialist partnerships: ANME-2c or ANME-1b with Desulfosarcinaceae/Desulfobulbales, embedded within modules that also include fermentative bacteria (e.g. Fermentibacterales and Anaerolineales) and polymer degraders [58–60]. At methane active, highly porous carbonate sites, but with higher organic carbon content [61], AOM consortia were present suggesting recycling of heterotrophic carbon. Low-methane active site carbonates were enriched in metabolically versatile taxa (e.g. Methylococcales, Thioglobaceae, Nitrosopumilales, and Kiloniellales) which can substitute electron acceptors (O_2_, NO_3_^−^) and partition niches under more oxic, nitrate-rich conditions. Genes linked to biofilms and quorum sensing were widespread at these sites, providing a basis for microbial interactions. Genome-scale modeling revealed a strong trade-off between predicted resource overlap (MRO) and cooperation potential (MIP), but no clear regional stratification. However, MAG-based analyses provide some empirical insights: Anaerolineales encode a branched-chain amino acid transporter (*livF/G/H/M/K*) which was more abundant at active methane sites, consistent with potential amino acid uptake and aligning with model-predicted metabolite exchange.

Our results based on incubation experiments are congruent with these model- and MAG-based site-specific differences considering their important constraints. For the incubations at active methane site carbonates, anoxic treatments were dominated by ANME and SRB at sampling endpoints, consistent with specialist AOM coupling. After oxygen addition, ^13^C-DIC production declined, but not significantly. Because oxygen concentrations were not tracked and only endpoint relative 16S profiles are available, we are unable to partition aerobic versus anaerobic contributions or infer growth of specific taxa. The oxygen-amended incubation of the low methane active carbonates exhibited ^13^C-DIC production rates higher than all other treatments. However, all other pairwise differences were not significant. And the relative abundance of heterotrophic bacteria at the culture endpoint was high. This suggests a strong stimulation of methane oxidation and DIC production by oxygen under low methane activity.

## Conclusions

The ecological functions of carbonate ecosystems are closely linked to the methane flux, shaping spatial differences in metabolic processes and likely also species interactions. High methane flux regions (e.g. lobster area) exhibit active AOM, facilitating carbon cycling, carbonate precipitation, and methane sequestration through a microbially driven self-sealing effect [14,15, 62–65]. Lower methane flux regions (e.g. the mussel area) show diminished AOM and communities enriched in ANME-1b relative to ANME-2c, with metabolism consistent with increased acetate production potential and reduced diagenetic efficiency [44]. Indicators such as consolidated macrobenthic shells, depleted carbon isotopes, and abundant aragonite in low_act carb sites carbonates indicate the realized natural succession of cold-seep ecosystems over time. Thus, our findings have revealed that microbiomes and their biogeochemical feedback loops play a significant role in shaping habitat-scale spatial heterogeneity of cold-seep carbonates.

## Supplementary Material

Supplementary_information_ycaf232

## Data Availability

The raw metagenomic sequencing data generated in this study have been deposited in the NCBI BioProject database under accession number PRJNA1061233 (https://www.ncbi.nlm.nih.gov/bioproject/PRJNA1061233).
