# Supplementary material for: Microbial communities and metabolic functions vary with spatial heterogeneity in cold-seep carbonates
Source: ISME Commun. 2025 Dec 11;5(1):ycaf232. doi: 10.1093/ismeco/ycaf232 (PMC12753302; doi:10.1093/ismeco/ycaf232)
Supplement: Supplementary_information_ycaf232 [file supplementary_information_ycaf232.pdf]

Microbial communities and metabolic functions vary with spatial heterogeneity in cold-seep carbonates

Manman Ma[1], Minxiao Wang[1,7#], Yue Liang[1], Yang Guo[1,4], Huan Zhang[1], Lei Cao[1], Lulu Fu[1], Gaowei Hu[5, 6], Chengfeng Li[5,6], Thomas Mock[1,3#] and Chaolun Li[1,2,7#]
[#]Corresponding authors

[1] Institute of Oceanology, Chinese Academy of Sciences, Qingdao, 266071, China.

[2] South China Sea Institute of Oceanology, Chinese Academy of Sciences, Guangzhou, 510301, China.

[3] School of Environmental Sciences, Univeristy of East Anglia, Norwich Research Park, Norwich, NR4 7TJ, United Kingdom.

[4] State Key Laboratory of Microbial Technology, Shandong University, Qingdao, 266237, China.

[5]. Key Laboratory of Gas Hydrate, Ministry of Natural Resources, Qingdao Institute of Marine Geology, Qingdao, 266237, China.

[6]. Laboratory for Marine Mineral Resources, Qingdao Marine Science and Technology Center, Laoshan Laboratory, Qingdao, 266237, China.

[7] University of Chinese Academy of Sciences, Beijing, 100049, China.

To whom correspondence should be addressed: wangminxiao@qdio.ac.cn; T.Mock@uea.ac.uk; lcl@qdio.ac.cn

SHORT TITLE: Microbiome in cold-seep carbonates

Methods

Microbial co-occurrence network analysis and sample-cluster association

ASV ssuRNA abundance tables, taxonomy, and sample metadata were combined in R (v4.3.1) using the phyloseq and ggClusterNet packages, with the full workflow available in Supplementary Methods (supplementary network.R). Networks were constructed by calculating SparCC correlations among taxa using corMicro (within ggClusterNet), followed by module detection using the Spinglass community detection algorithm (model_maptree). Each module ("graph") consists of highly interconnected taxa.

To associate modules with specific environments, for each module we calculated the cumulative abundance across all member taxa within each sample. The module was considered most strongly linked to the environment(s) where its cumulative abundance was highest ("RelativeContribution.pdf", output of pipeline). This quantitative approach ensures associations are data-driven and reproducible.

Visualizations were produced with ggplot2 and pheatmap in R, and full scripts are provided in the Supplementary Files.

Metabolic models for each MAG (GEMs) were constructed as follows.

GEMs were constructed using CarveMe v1.5.2 [1] with the Gurobi solver (v11.0.2) [2] , based on prokka-annotated protein FASTA files and default gapfilling parameters. Community-level metabolic interactions under minimal media were simulated using SteadierCom v1.2.0 (Daniel Machado, NTNU, 2024: https://github.com/cdanielmachado/SteadierCom). Predicted global metabolic interaction metrics, including metabolic resource overlap (MRO) and metabolic interaction potential (MIR), were computed using SMETANA [3] with default settings. The use of GEM/SMETANA-derived metrics to infer potential competition and metabolite exchange has been benchmarked or cross-validated in multiple systems [4-8].

The qPCR standard curves were constructed as follows.

Environmental DNA was first extracted, and the target gene was PCR-amplified. The PCR product was purified using a Gel Extraction Kit (Takara, Japan). The purified product was then ligated into the pMD19-T vector (Takara, Japan) and transformed into Escherichia

coli DH5α competent cells (TransGen Biotech, China) according to the manufacturer's instructions. Positive clones were screened by colony PCR and further confirmed by Sanger sequencing (Sangon Biotech, China). Plasmids containing the correct insert were extracted using a Plasmid Mini Kit (Omega Bio-Tek, USA). The plasmid DNA was quantified with a NanoDrop 2000 spectrophotometer (Thermo Scientific, USA), and ten-fold serial dilutions were prepared to generate the standard curve (Fig. S1) for absolute quantification in qPCR.

16S rRNA Gene Amplicon Sequencing and Analysis

For each incubation experiment, three biological replicates were used. DNA was extracted directly from the post-incubation carbonate or sediment samples. The V3–V4 regions of the bacterial 16S rRNA gene were amplified with primers 341F and 806R; archaeal communities were profiled using primers Arch519F and Arch915R.

PCR products were pooled and purified using a Qiagen gel extraction kit, with library preparation performed using the NEBNext® Ultra™ II DNA Library Prep Kit. Libraries were sequenced on an Illumina NovaSeq6000 platform.

Raw sequences were split by barcode, merged, quality-filtered, and chimeras removed as previously described. ASVs were inferred with DADA2 in QIIME2, and singletons or low-abundance ASVs (frequency <5) were discarded. Taxonomic assignment was performed against the SILVA database (v138.1).

Alpha and beta diversity analyses were performed with QIIME2, with rarefaction applied to normalize sequencing depth where appropriate.

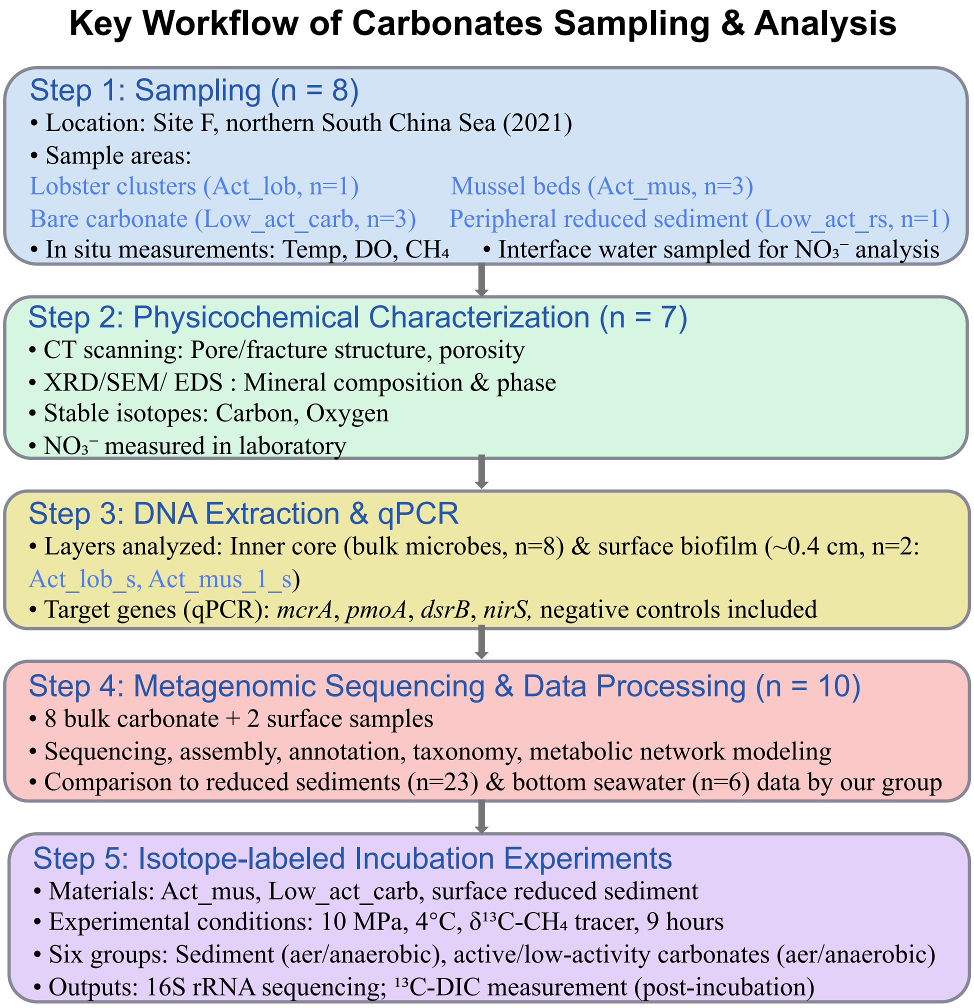

Fig. S1 Overview of study design and workflow

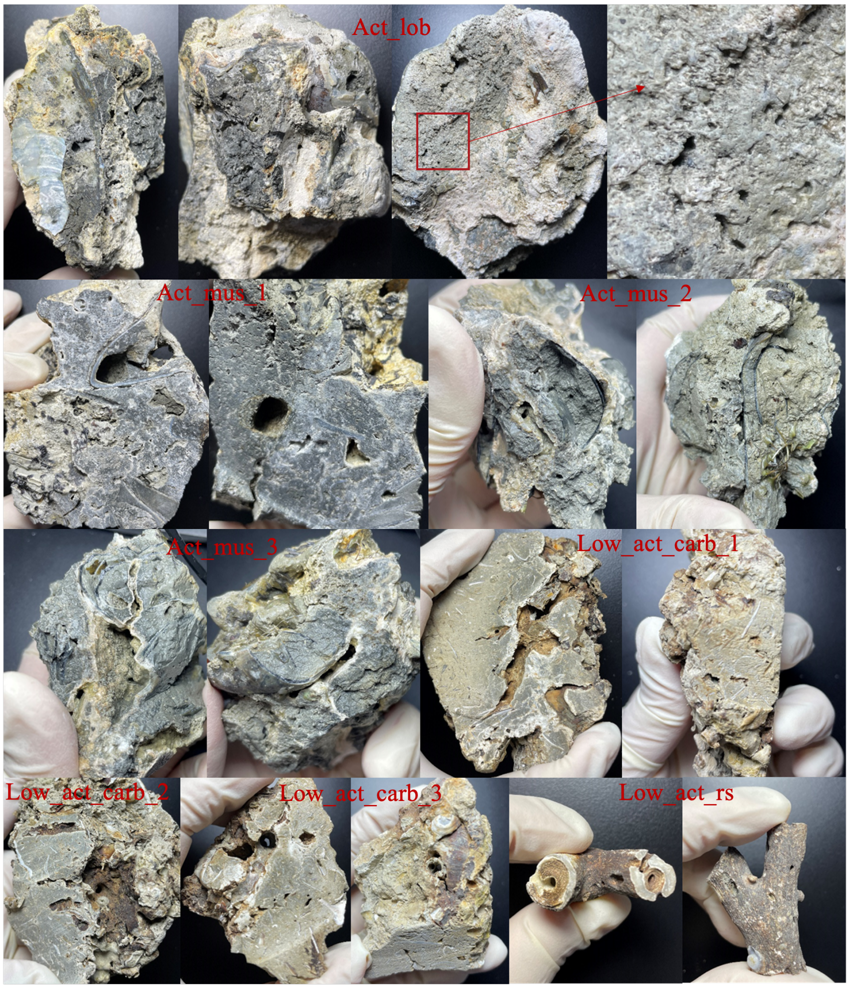

Fig. S2 Pictures of the carbonate samples.

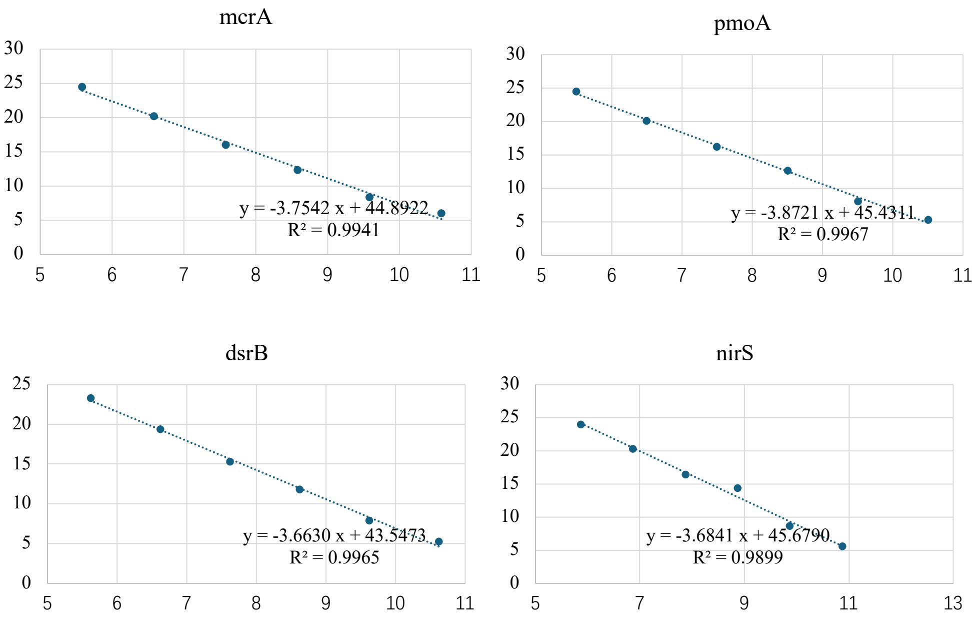

Fig. S3 Standard curves for quantitative analysis of *mcrA, pmoA, dsrB*, and *nirS* genes.

Table S1 Primers used in this study.

Target gene	Primer	Sequence (5'-3')	Reference
mcrA gene	mlas	GGTGGTGTMGGDTTCACMCARTA	[9]
	mcrA rev	CGTTCATBGCGTAGTTVGGRTAGT	
pmoA gene	A189F	GGNGACTGGGACTTCTGG	[10, 11]
	mb661R	CCGGMGCAACGTCYTTACC	
dsrB gene	DSRp2060F	CAACATCGTYCAYACCCAGGG	[9]
	DSR4R	GTGTAGCAGTTACCGCA	
nirS gene	cd3aF	GTSAACGTSAAGGARACSGG	[9]
	R3cd	GASTTCGGRTGSGTCTTGA	

Table S2 Environmental parameters of bottom seawater – carbonate interface and environmental background of carbonate habitat.

	temp (◦C)	CH_4 (μM)	DO (mg/L)	NO_3^- (μM)	SO_4^{2-} (mM)*	Aragonite (%)	Quartz (%)	Calcite (%)	d $^{13}C/^{12}C$ vs VPDB(‰)	d $^{18}O/^{16}O$ vs VSMOW(‰)	porosity (%)
Act_lob	3.59	8.10	0.1~1.5	17.37	28	89	8	3	-44.71	4.48	11.561
Act_mus_1	3.69	1.58	0.8~3.1	22.32	28	87	12	1	-47.10	4.64	13.121
Act_mus_2						89	5	7	-52.28	4.86	11.768
Act_mus_3						84	15	0	-41.81	4.68	9.559
Low_act_carb _1	3.65	0.38	2.5~3.0	24.20	28	84	12	4	-40.23	4.65	9.178
Low_act_carb _3						84	14	2	-46.43	4.78	6.012
Low_act_rs	3.63	0.055	3.0~3.2	21.26	28	0	1.56	98.44	-3.32	2.77	12.335

* Take the high sulfate concentration (28 mM) in seawater as the environment background value, which makes it the most abundant and, often, important electron acceptor at cold seeps [12].

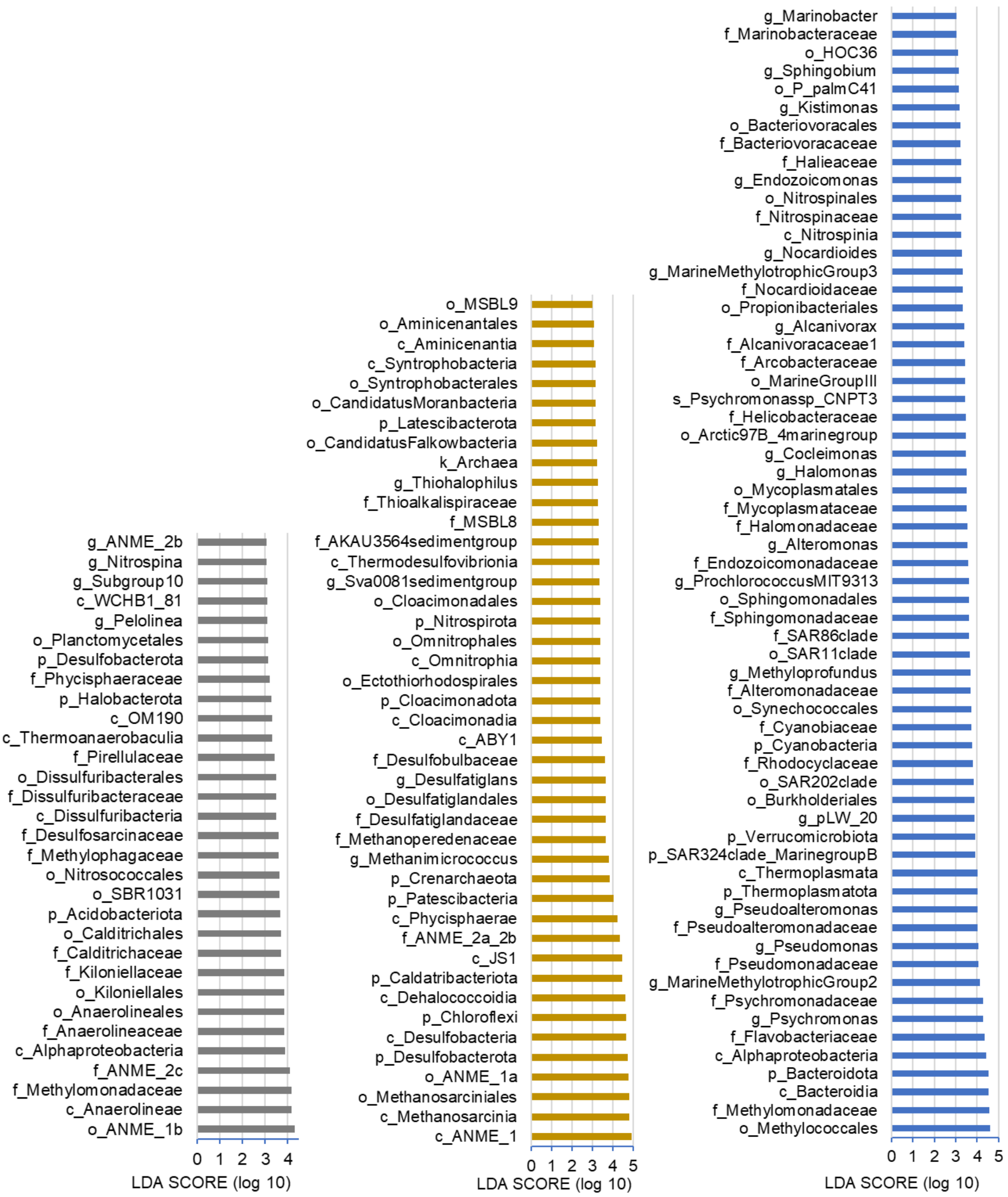

Fig. S4 Biomarker microbes in different habitats based on an LDA score of 3. Gray: carbonate; yellow: reduced sediment; blue: bottom seawater.

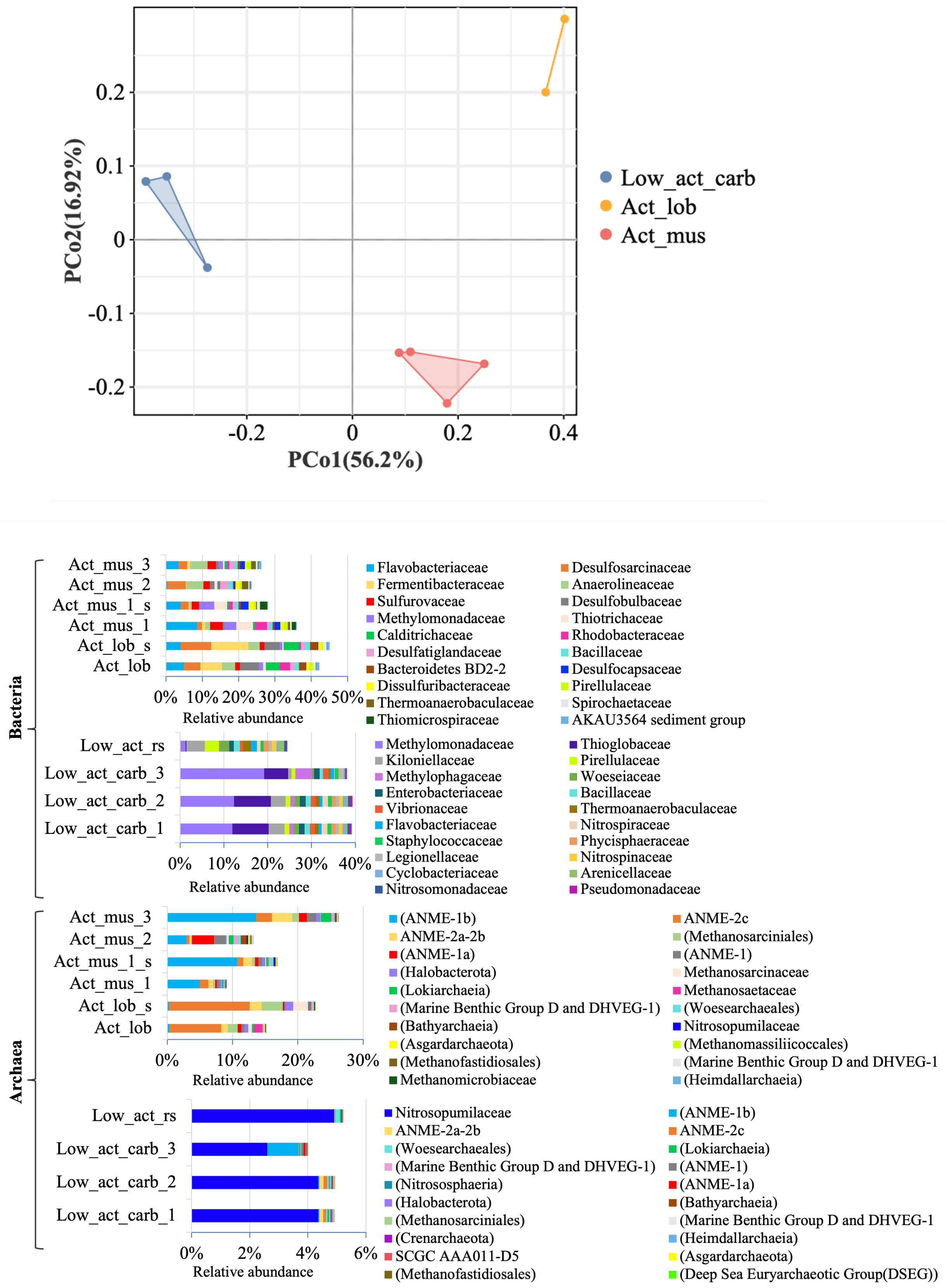

Fig. S5 PCoA analysis of microbial communities in carbonate habitats of different areas (top). Taxa of bacteria and archaea at family level in carbonate habitats (bottom).

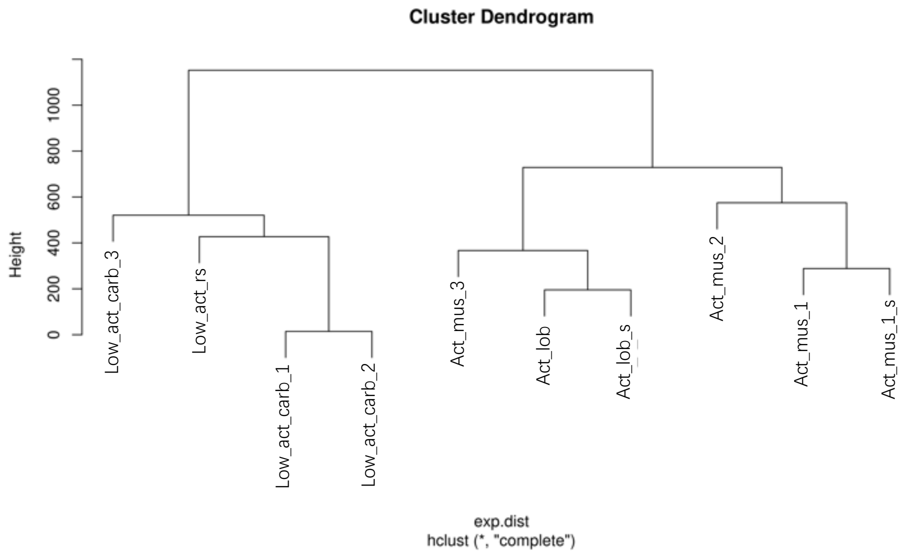

Fig. S6 Cluster dendrogram of key genes participate in carbon, nitrogen, and sulfur metabolisms.

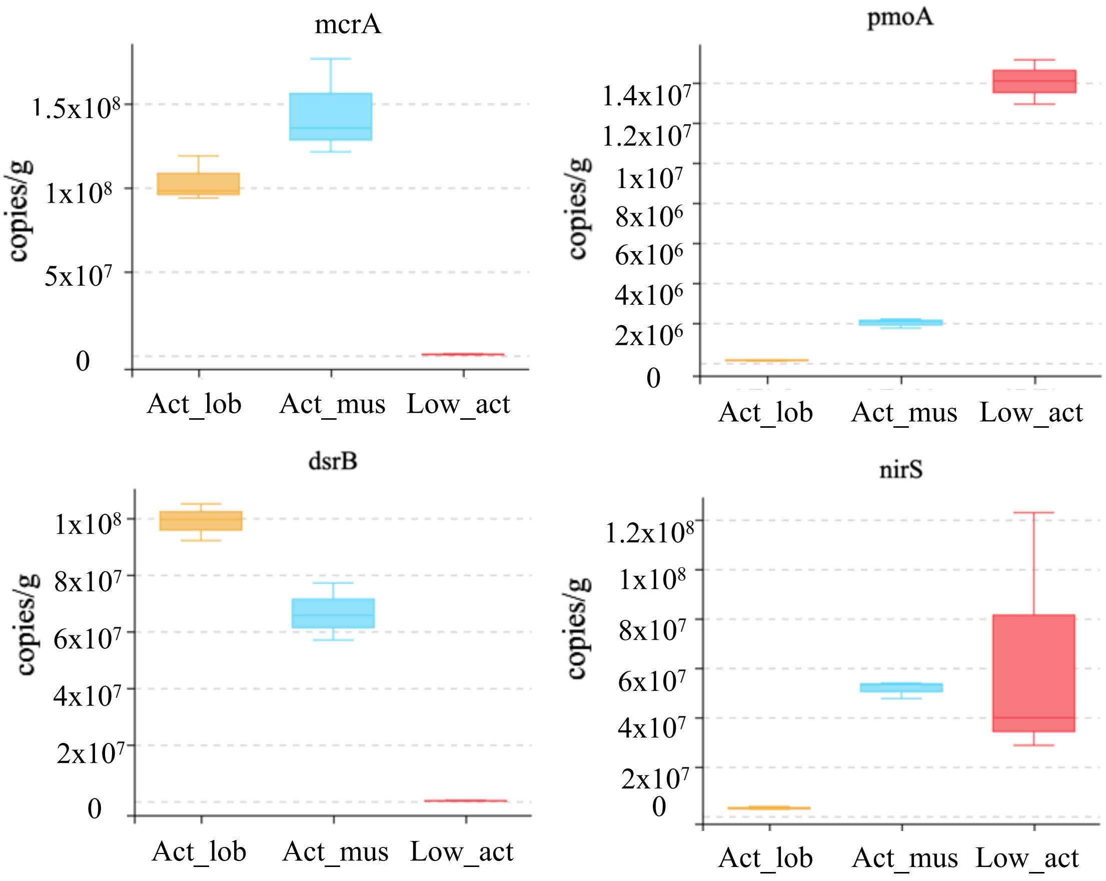

Fig. S7 QPCR results based on *mcrA*, *pmoA,dsrB and nirS* genes.

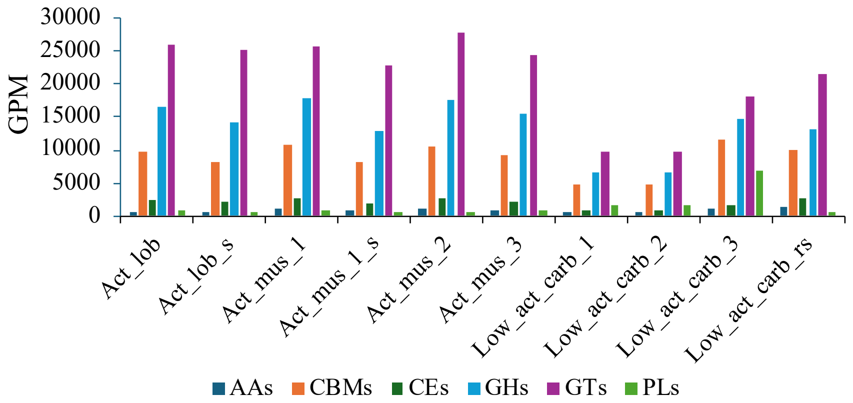

AAs: Auxiliary Activities; CBMs: Carbohydrate-Binding Modules;
CEs: Carbohydrate Esterases; GHs: Glycoside Hydrolases;
GTs: GlycosylTransferases; PLs: Polysaccharide Lyases

Fig. S8 Distribution characteristics of carbohydrate active enzymes in carbonate habitats.

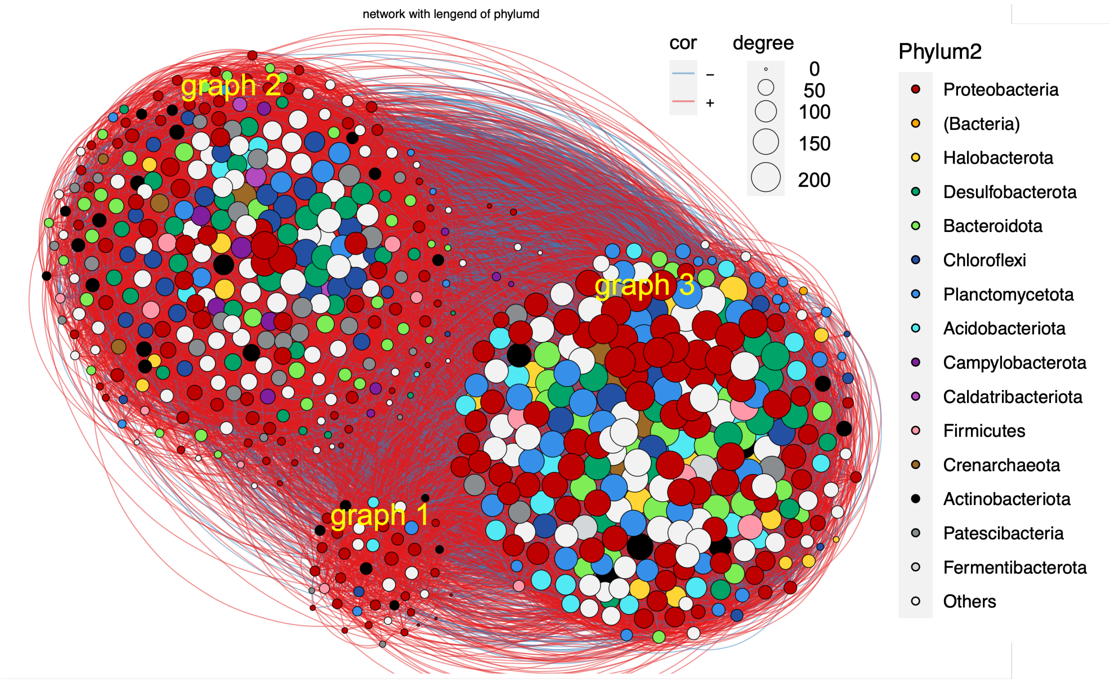

Fig. S9 Microbial co-occurrence network colored by phylum. Each node represents a microbial taxon, colored by phylum (see legend). Node size indicates the “degree,” i.e., the number of direct connections (edges) with other nodes in the network (degree centrality). Edges represent significant correlations (red for positive, blue for negative). Larger nodes correspond to taxa with higher degree, indicating more direct associations. Three network modules (“graph 1–3”) are highlighted. The correlation type (positive/negative) and phylum-level color code are indicated in the legend.

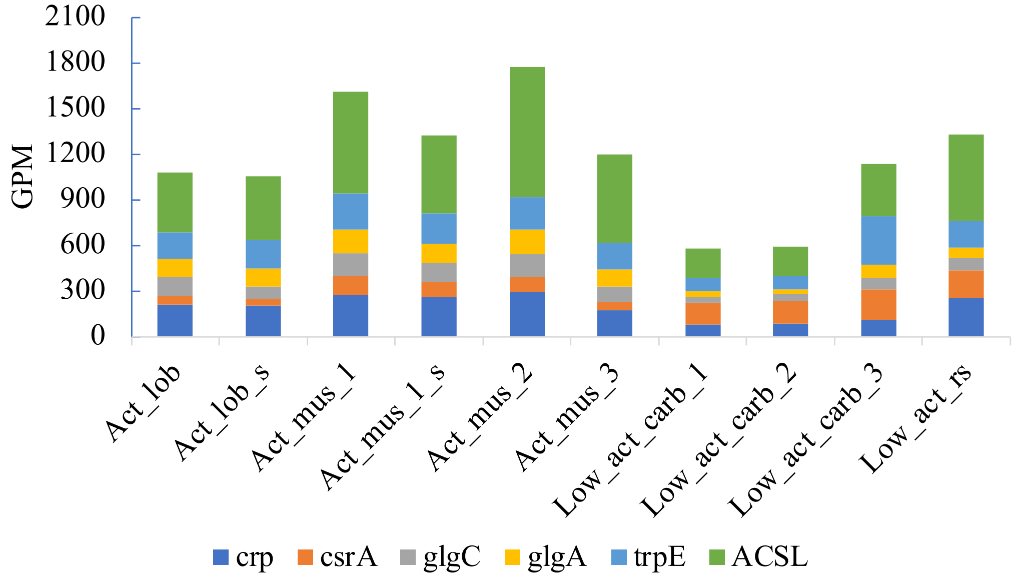

Fig. S10 Abundance of genes related to biofilm formation and quorum sensing.

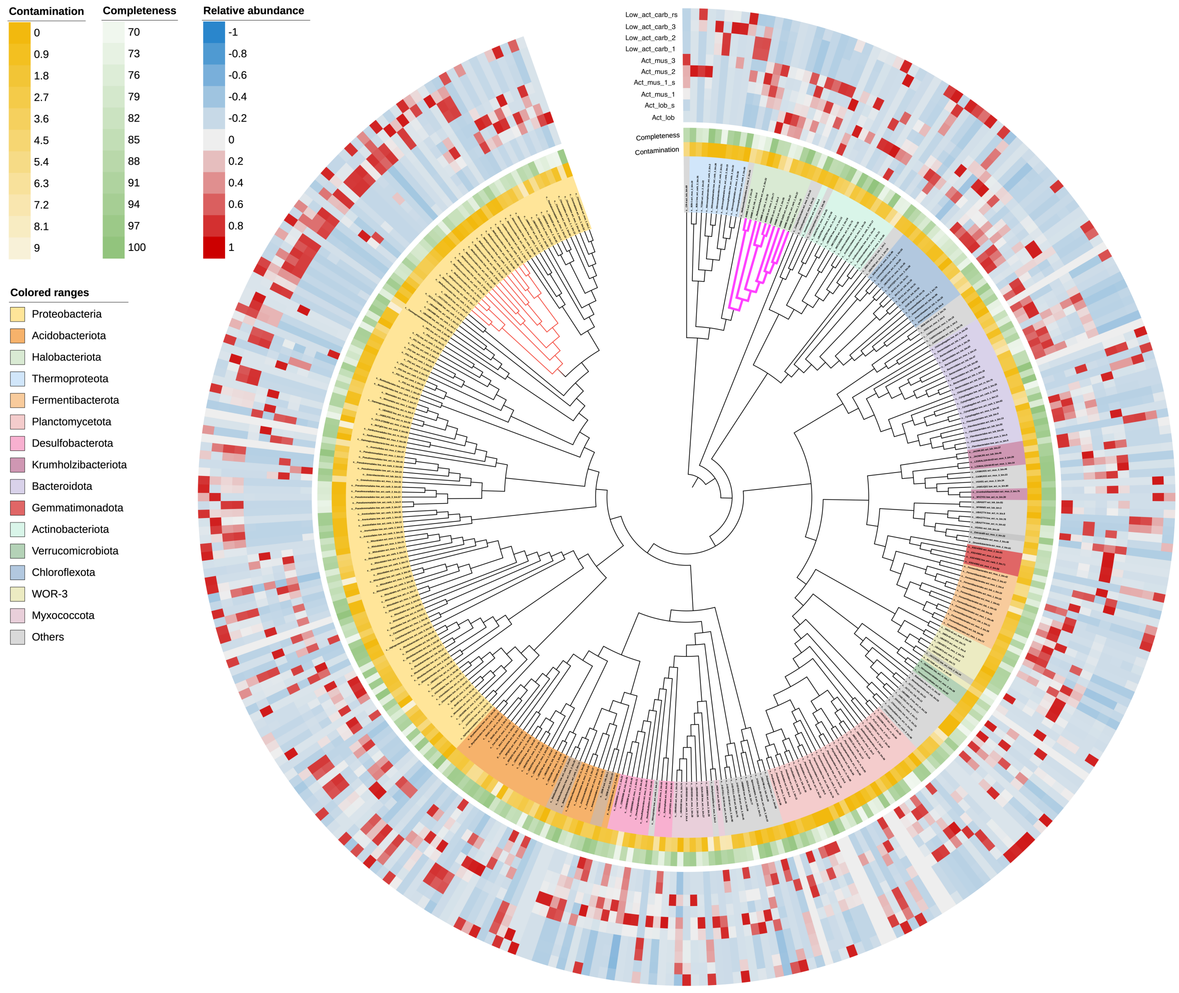

Fig. S11 MAGs with completeness greater than 70%, contamination less than 10%.

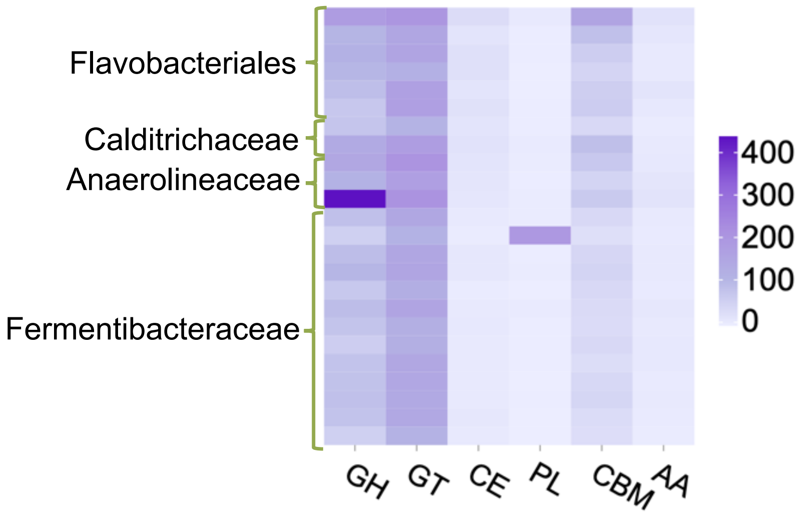

Fig. S12 Carbohydrate active enzymes distribution in MAGs of *Flavobacteriales, Calditrichaceae, Anaerolineaceae* and *Fermentibacteraceae*.

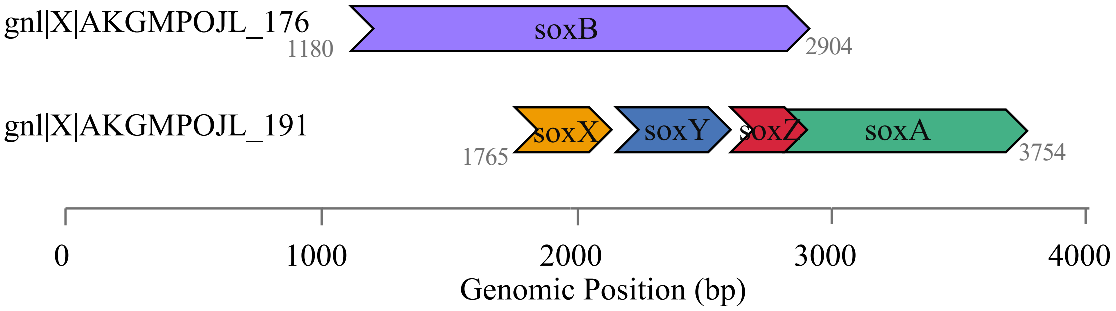

Fig. S13 Gene organization of sox gene clusters in Methylococcales MAG from low-activity carbonate samples

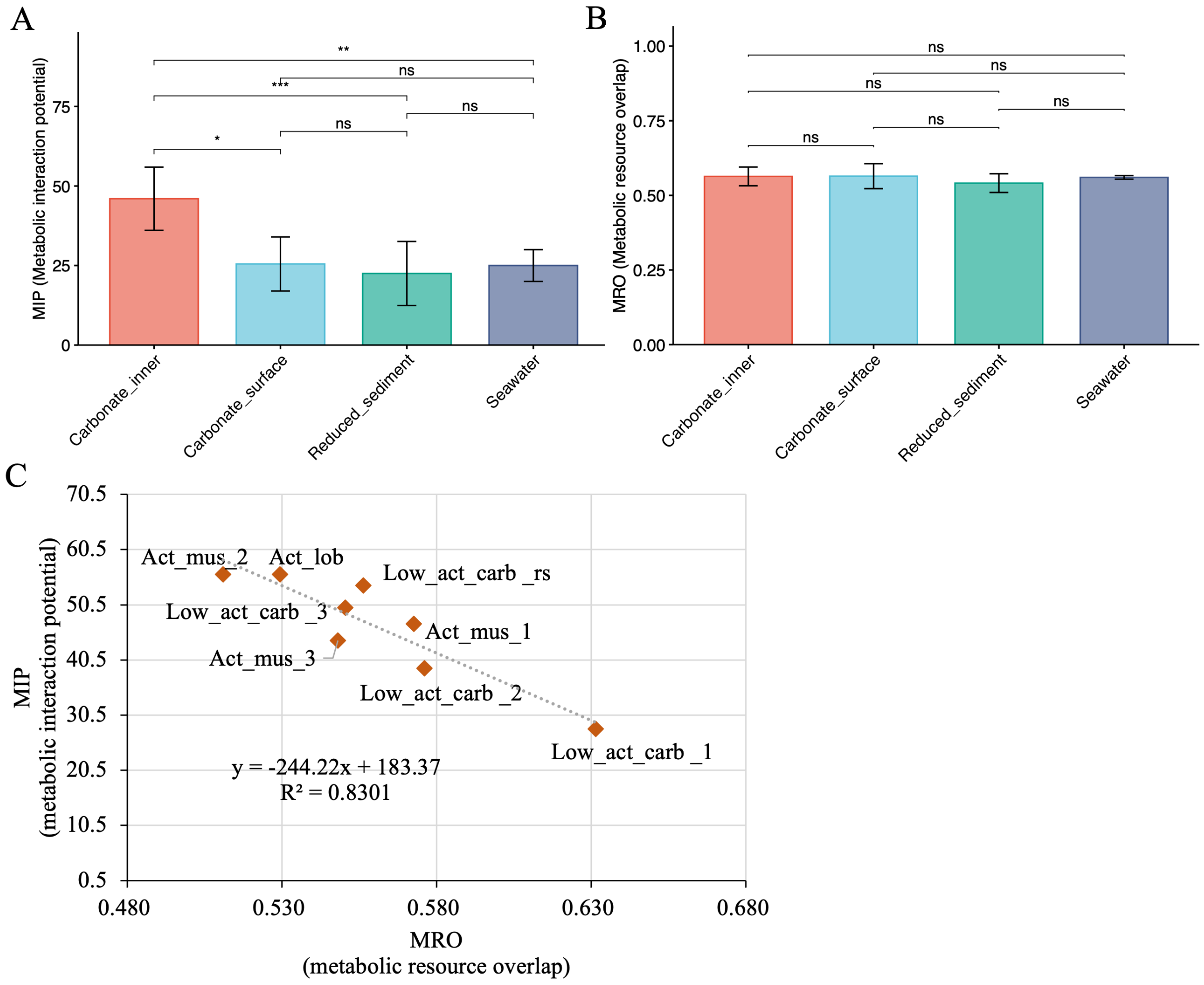

Fig. S14 Competition (MRO) and cooperation (MIP) scores in different habitats (A, B) and the correlation between MIP and MRO of inner carbonate samples (C). ns: not statistically significant.

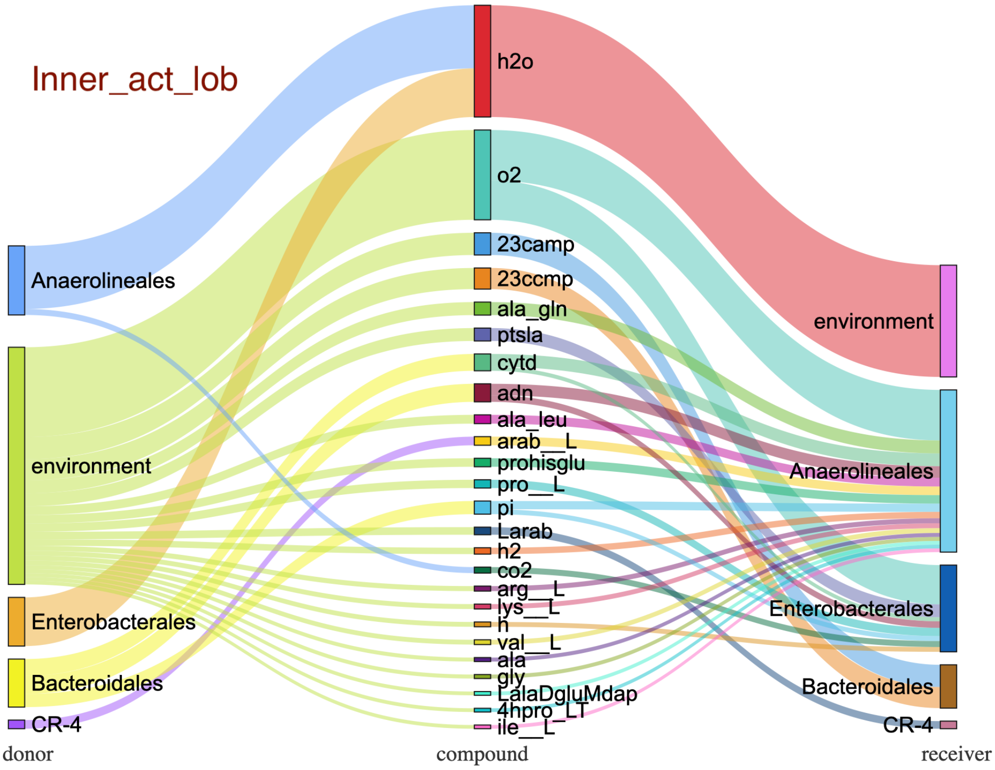
Inner_act_lob
Anaerolineales
environment
Enterobacterales
Bacteroidales
CR-4
donor
h2o
o2
23camp
23ccmp
ala_gln
ptsla
cytd
adn
ala_leu
arab__L
prohisglu
pro__L
pi
Larab
h2
co2
arg__L
lys__L
h
val__L
ala
gly
LalaDgluMdap
4hpro_LT
ile__L
compound
environment
Anaerolineales
Enterobacterales
Bacteroidales
CR-4
receiver

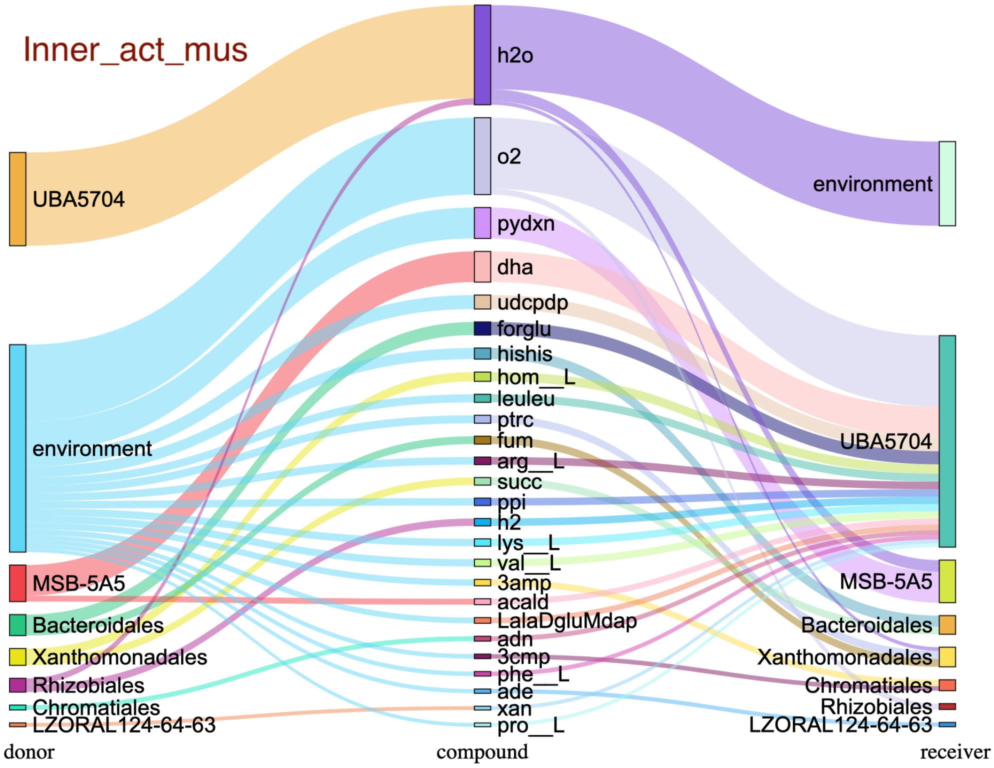
Inner_act_mus
UBA5704
environment
MSB-5A5
Bacteroidales
Xanthomonadales
Rhizobiales
Chromatiales
LZORAL124-64-63
donor
h2o
o2
pydxn
dha
udcpdp
forglu
hishis
hom__L
leuleu
ptrc
fum
arg__L
succ
ppi
h2
lys__L
val__L
3amp
acald
LalaDgluMdap
adn
3cmp
phe__L
ade
xan
pro__L
compound
environment
UBA5704
MSB-5A5
Bacteroidales
Xanthomonadales
Chromatiales
Rhizobiales
LZORAL124-64-63
receiver

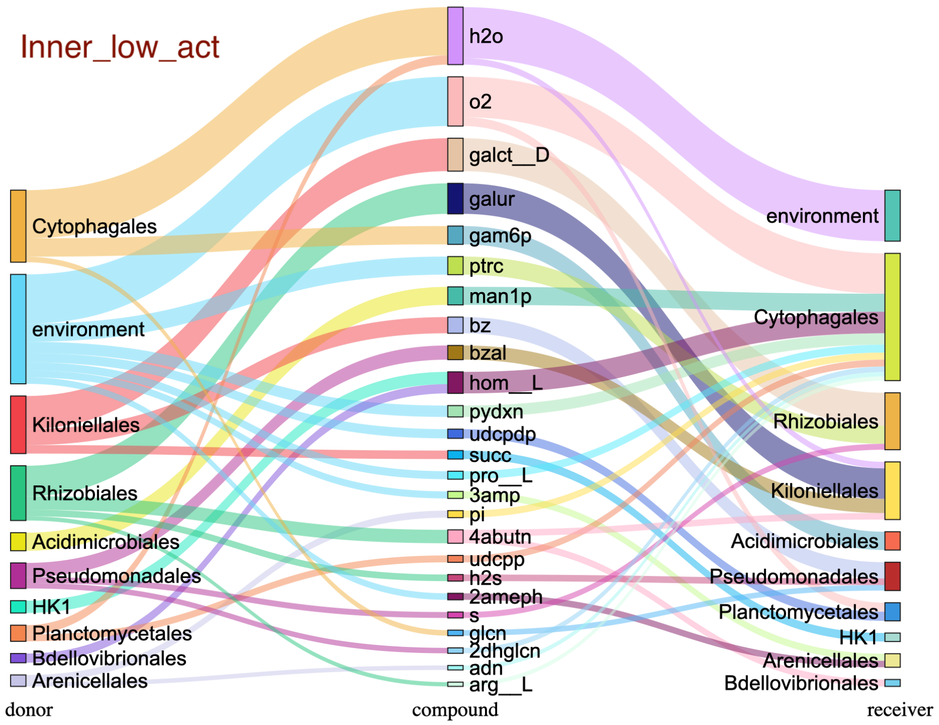

Fig. S15 Alluvial diagrams showing compounds exchanged (mass rate: top 30) between the donor and receiver. The compound name is an abbreviation in the bigg database (http://bigg.ucsd.edu).

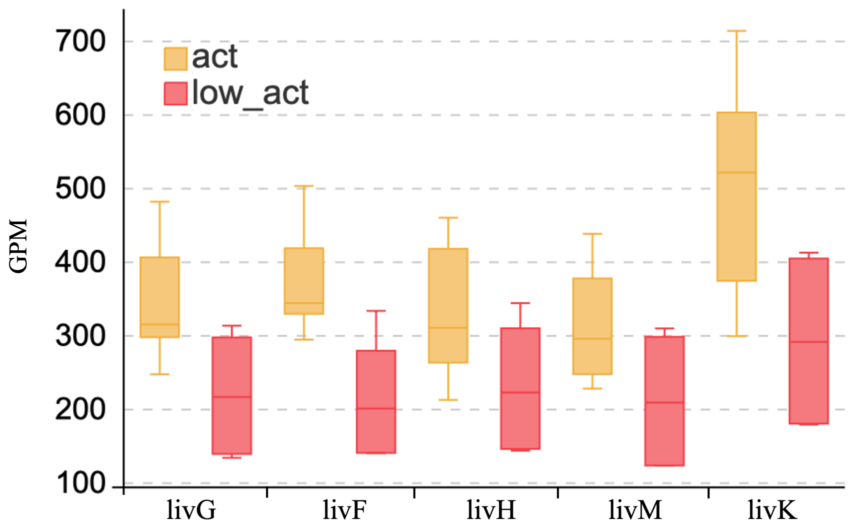

Fig. S16 Abundance distribution of liv operon genes (*livF/G/H/M/K*) in active and low-activity regions. *livG*: branched-chain amino acid transport system ATP-binding protein; *livF*: branched-chain amino acid transport system ATP-binding protein; *livH*: branched-chain amino acid transport system permease protein; *livM*: branched-chain amino acid transport system permease protein; *livK*: branched-chain amino acid transport system substrate-binding protein.

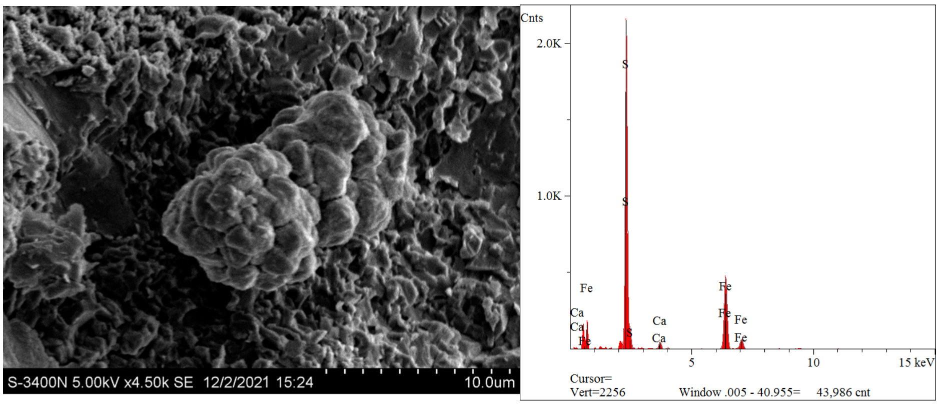

Fig. S17 Pyrite detected in carbonate samples: SEM (Left), EDS (right).

Table S4 Alpha diversity indices of microbial communities at the species and gene levels across different sampling sites.

Sample	Species diversity			Gene diversity		
	Shannon	Pielou	SR	Shannon	Pielou	SR
Act_lob	4.9184	0.6581	1762	2.915	0.327	7396
Act_lob_s	4.506	0.6518	1005	2.894	0.327	6959
Act_mus_1	5.1964	0.6621	2561	3.653	0.406	8070
Act_mus_1_s	5.0311	0.6815	1607	3.126	0.35	7478
Act_mus_2	5.2583	0.6699	2564	3.482	0.389	7780
Act_mus_3	5.1111	0.6553	2440	3.075	0.342	7956
Low_act_carb_1	4.33	0.5895	1549	1.452	0.162	7618
Low_act_carb_2	4.2976	0.5781	1693	1.48	0.166	7640
Low_act_carb_3	3.6944	0.4808	2172	2.692	0.3	7948
Low_act_rs	5.027	0.6583	2073	2.845	0.317	7943

Reference

1. Machado, D., S. Andrejev, M. Tramontano,K.R. Patil. Fast automated reconstruction of genome-scale metabolic models for microbial species and communities. Nucleic acids research, 2018. 46(15): 7542-7553.

2. Chiciudean, I., G. Russo, D.F. Bogdan, E.A. Levei, L. Faur, A. Hillebrand-Voiculescu, et al. Competition-cooperation in the chemoautotrophic ecosystem of Movile Cave: first metagenomic approach on sediments. Environmental microbiome, 2022. 17(1): 44-61.

3. Zelezniak, A., S. Andrejev, O. Ponomarova, D.R. Mende, P. Bork,K.R. Patil. Metabolic dependencies drive species co-occurrence in diverse microbial communities. Proceedings of the National Academy of Sciences of the United States of America, 2015. 112(20): 6449-6454.

4. Machado, D.O.M. MaistrenkoS. AndrejevY. KimP. BorkK.R. Patil, et al. Polarization of microbial communities between competitive and cooperative metabolism. Nature Ecology & Evolution, 2021. 5(2): 195-203.

5. Wendering, P.,Z. Nikoloski. COMMIT: Consideration of metabolite leakage and community composition improves microbial community reconstructions. PLOS Computational Biology, 2022. 18(3): e1009906.

6. Marcelino, V.R., C. Welsh, C. Diener, E.L. Gulliver, E.L. Rutten, R.B. Young, et al. Disease-specific loss of microbial cross-feeding interactions in the human gut. Nature Communications, 2023. 14(1): 6546.

7. Lecomte, M.W. CaoJ. AubertD.J. ShermanH. FalentinC. Frioux, et al. Revealing the dynamics and mechanisms of bacterial interactions in cheese production with metabolic modelling. Metabolic Engineering, 2024. 83: 24-38.

8. Wang, D., K.A. Hunt, B. Abrahamson, Z. Flinkstrom, X. Tao, R.S. Tanner, et al. Higher-order microbial interactions revealed by comparative metabolic modeling of synthetic communities with varying species composition. ISME Communications, 2025. 5(1).

9. Niu, M., F. Xibei, G.-c. Zhuang, Q. Liang,F. Wang. Methane-metabolizing microbial communities in sediments of the Haima cold seep area, northwest slope of the South China Sea. FEMS Microbiology Ecology, 2017. 93.

10. Costello, A.M.,M.E. Lidstrom. Molecular characterization of functional and phylogenetic genes from natural populations of methanotrophs in lake sediments. Applied and environmental microbiology, 1999. 65(11): 5066-5074.

11. Holmes, A.J., A. Costello, M.E. Lidstrom,J.C. Murrell. Evidence that participate methane monooxygenase and ammonia monooxygenase may be evolutionarily related. FEMS Microbiology Letters, 1995. 132(3): 203-208.

12. Joye, S.B. The Geology and Biogeochemistry of Hydrocarbon Seeps. Annual Review of Earth and Planetary Sciences, 2020. 48: 205-231.
